# Complex‐mediated nucleophilic aromatic substitution with aryl nitriles to realize intramolecular flapping‐restricted D‐A AIEgens for bioimaging

**DOI:** 10.1002/smo.20240039

**Published:** 2024-11-14

**Authors:** Feng Liu, Junkai Liu, Junyi Gong, Runfeng Lin, Siyuan Qiu, Zicheng Liu, Chongyang Li, Miao Meng, Shijie Li, Mei Tu, Jacky W. Y. Lam, Guangle Niu, Ming Chen

**Affiliations:** ^1^ College of Chemistry and Materials Science Jinan University Guangzhou China; ^2^ Department of Chemistry Hong Kong Branch of Chinese National Engineering Research Center for Tissue Restoration and Reconstruction The Hong Kong University of Science and Technology Kowloon Hong Kong China; ^3^ School of Science and Engineering Shenzhen Institute of Aggregate Science and Technology The Chinese University of Hong Kong, Shenzhen (CUHK-SZ) Shenzhen Guangdong China; ^4^ Clinical Medical College of Acupuncture Moxibustion and Rehabilitation Guangzhou University of Chinese Medicine Guangzhou China; ^5^ School of Chemistry and Chemical Engineering Beijing Institute of Technology Beijing China

**Keywords:** aggregation‐induced emission, bioimaging, donor‐acceptor structure, nucleophilic aromatic substitution

## Abstract

Donor‐acceptor (D‐A) compounds are particularly important in optoelectronic and biological applications. However, they are normally synthesized in the presence of transition metal catalysts. Herein, we report a metal‐free method by a complex‐mediated nucleophilic aromatic substitution of aryl nitriles with amines. The method can lead to rich D‐A type aggregation‐induced emission luminogens (AIEgens) with tunable properties. They emit from deep‐blue to yellow‐green and possess high photoluminescence quantum yields up to 70.5% in the aggregate state. Interestingly, the suppression of intramolecular flapping is proved to play an indispensable role in the AIE behavior, which is different from the mechanism met in other AIEgens. Moreover, the biocompatible AIEgens possess specific staining of lipid droplets in HeLa cells and the superiority of identifying fatty liver over traditional Oil Red O staining is exhibited.

## Graphical abstract

In this work, we developed a complex‐mediated nucleophilic aromatic substitution which can generate rich D‐A AIEgens under a facile condition. The AIE mechanism of such AIEgens was unveiled and their application towards fatty liver imaging was exhibited.

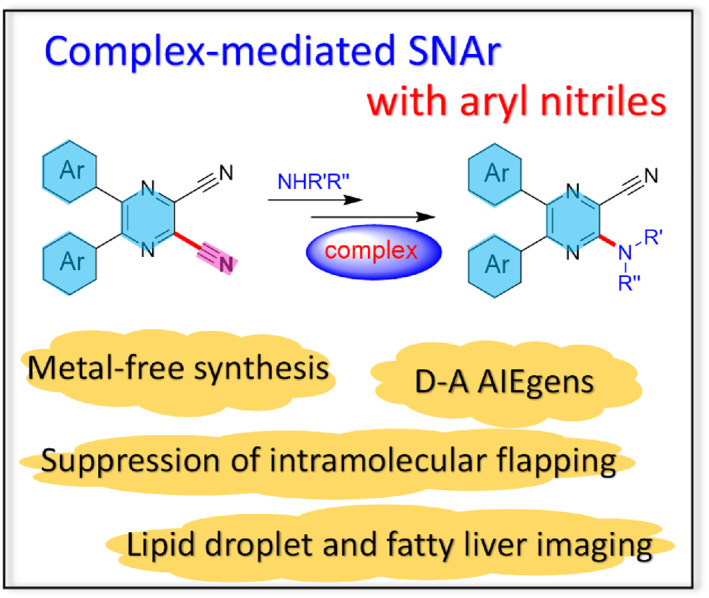

## 1 INTRODUCTION

Conjugated compounds with donor‐acceptor (D‐A) architecture have been paid massive attention in optoelectronic and biological applications due to their tunable electronic property.^[1]^ On the other hand, fluorescent molecules with aggregation‐induced emission (AIE) characteristic have shown great promise in practical applications due to their intrinsic behavior to overcome the aggregation‐caused quenching effect and the high luminescent efficiency in the aggregate state.^[2]^ Currently, endowing the AIE luminogens (AIEgens) with D‐A effect have been regarded as an ideal strategy to regulate the energy gap or singlet‐triplet splitting of the molecules, which enables them to realize the various applications such as organic light‐emitting diodes, bioimaging and biotherapy, etc.^[3]^ Up to now, a large amount of D‐A AIEgens have been prepared, while their synthetic approaches still focus on Buchwald‐Hartwig amination reaction, Suzuki‐Miyaura cross‐coupling reaction, and Sonogashira coupling reaction, etc.^[4]^ These reactions always require transition metal catalysts, therefore showing apparent toxicity and increased cost. Besides, most of them are carried out under basic and inert atmosphere. These factors complicate the preparation process and inevitably cause a limitation to the large‐scale application. Thus, the development of a facile synthetic methodology towards D‐A AIEgens not only possesses marvelous academic values but also is particularly useful to accelerate the practical applications.

Aryl nitriles are common substances and widely found in natural products, drugs, and optoelectronic materials.^[5]^ The existence of cyano groups plays a vital role in affecting their properties. For example, it is helpful to improve the drug activity by its increased interaction with proteins.^[6]^ On the other hand, the strong electron‐withdrawing property of the cyano group facilitates the regulation of the electronic properties of conjugated molecules, rendering them as active materials in organic light‐emitting diodes, organic solar cells, organic field‐effect transistors, and biological applications.^[7]^ Meanwhile, the cyano group, which is regarded as one of the triple bond groups, shows evident chemical reactivity due to its unsaturated and unique electronic properties. This enables aryl nitrile compounds to be widely used as intermediates in the synthetic chemistry. Nevertheless, the current reactions are still based on nucleophilic addition of cyano groups to generate carboxyl, benzylamine, amide and ketone groups or cyclization reactions to form heterocycles such as triazine, tetrazine, thiadiazole and oxazole.^[8]^ Direct substitution of cyano groups by C‐CN bond cleavage on aryl cores is still challenging and highly desirable for an in‐depth investigation.

**FIGURE 1:**
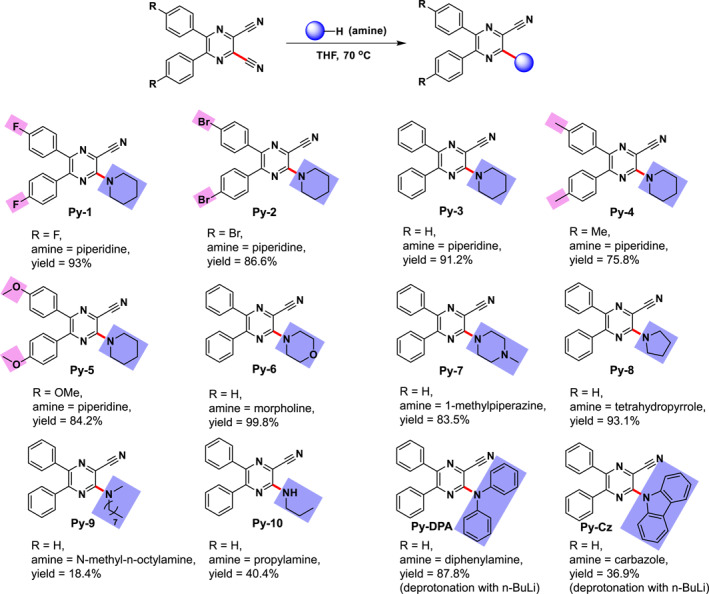
Substitution of the cyano group in pyrazine‐based aryl nitriles with amines to generate different products.

In this work, we develop a facile method to prepare D‐A AIEgens by a complex‐mediated nucleophilic aromatic substitution of pyrazine‐based aryl nitriles with amines under a mild condition (Figure 1). The reaction is free from the transition metal catalyst and possesses the merits of high efficiency, good yield and wide substrate universality, etc. The resulting D‐A AIEgens show tunable photophysical properties decided by the substituents and high luminescence yields up to 70.5% in the aggregate state. Interestingly, it is revealed that the suppression of intramolecular flapping plays an indispensable role in the AIE behavior. Moreover, the AIEgens also show good biocompatibility and specific lipid droplet staining ability in HeLa cells, and the further superiority to identify the fatty liver over traditional Oil Red O staining is demonstrated. The significance of our work is as follows: (1) the substitution of cyano groups is always not involved in the scope of nucleophilic aromatic substitution, however, our study reveals a rare complex formation between the aryl nitriles and amines to promote the self‐catalyzation to benefit the reaction, therefore making the impossible possible; (2) the reaction can proceed efficiently without transition metal catalyst under a mild condition, which is economic, environmentally friendly, and suitable for a large‐scale production; (3) besides the excellent AIEgens, the AIE mechanism reported in this work is different from the previous ones (e.g., restriction of intramolecular phenyl rotation or double bond twistion), therefore providing a platform to tune the property of luminescent materials; (4) compared with traditional method for fatty liver staining, the fluorescent imaging with our AIEgen is handy and shows better sensitivity, which can serve as a useful tool for further diagnosis of fatty liver disease from a liver biopsy. We believe that with this work more D‐A compounds will be sparked based on this facile method to promote the practical applications.

## 2 RESULTS AND DISCUSSION

During our course of synthesizing organic optoelectronic materials, we attempted to carry out a Sonogashira reaction between dibromo‐substituted 2,3‐dicyano‐5,6‐diphenylpyrazine (**2**) and trimethylsilylacetylene to generate the silylacetylene derivative (**2′**), while Pd(PPh_3_)_2_Cl_2_, CuI, PPh_3_, triethylamine and piperidine were used as catalysts, ligand and bases, respectively. However, the reaction can only lead to an unexpected product by replacing one of its cyano groups with piperidine in a very high yield, with its structure confirmed by the single crystal diffraction (Scheme S1). It implies that the aryl nitrile may undergo an undesired but active substitution reaction by a C‐CN bond cleavage. This motivates us to give a systematic investigation of this reaction.

To exclude the influence from the bromine substituents, we conducted the reaction with 2,3‐dicyano‐5,6‐diphenylpyrazine (**3**) under the same conditions described above. We find that the reaction proceeds smoothly to afford the substituted product efficiently. The reaction condition is further simplified. For example, the reactions can finish in the absence of the catalysts, ligand and triethylamine and it is concluded that these factors exert no effect on the reaction. Moreover, the reaction can work pleasantly in the air atmosphere without inert protection due to its metal‐free condition. The more specific investigation indicates that the reaction ends within 18 h in the air in the presence of piperidine (1.2 equiv.) in THF at reflux and furnishes the product in a high yield of 91.2% after purification by a chromatographic column (Figure 1 and Table S1). When the hydrogen atoms in phenyl rings of **3** are replaced from electron‐withdrawing fluorine and bromine atoms to electron‐donating methyl and methoxyl groups, the reaction shows a similar efficiency, indicating that the reaction reactivity is less affected by these substituents (Figure 1). The probability of this reaction in the presence of other amines including primary amines, secondary amines and aryl amines are also investigated. As piperidine is replaced with morpholine, 1‐methylpiperazine, and tetrahydropyrrole (regarded as heteroatom‐substituted or ring‐contracted derivatives of piperidine), the expected products can still be obtained in high yields (Figure 1). The reaction still proceeds with a long alkyl amine (e.g., N‐methyl‐*n*‐octylamine), though the bulk effect of the alkyl chain obviously affects the yield (Figure 1). Besides the aliphatic secondary amines, the primary amine is applicable in the reaction but has a lower yield due to its weaker alkalinity (Figure 1). Carrying out the reaction with aryl amines (e.g., diphenylamine and carbazole) is difficult due to their bad nucleophilicity decided by the increased interaction between aromatic ring and nitrogen atom. However, this problem can be overcome by improving the nucleophilicity of aryl amines through deprotonation with *n*‐BuLi (Figure 1, Scheme S2 and Table S1). Note that most of these reactions can finish within 24 h with 1.2 equivalent of amines, while the others need more amines to accelerate the reaction (Table S1). Moreover, the introduction of heteroatoms into amines and the employment of bulky and primary amines may pose some negative effects on the reactions. All these suggest that the method developed by us is bestowed with merits of simple and mild reaction conditions, high efficiency, excellent yield and wide amine universality, etc.

**FIGURE 2:**
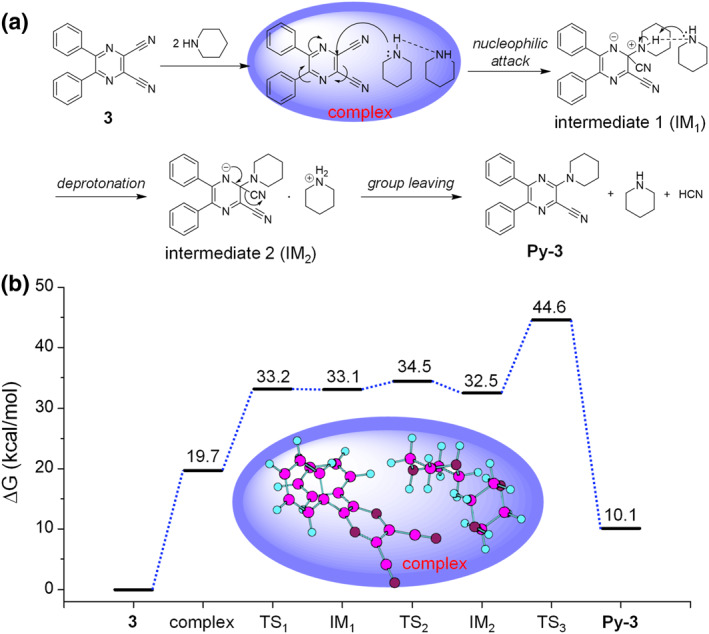
(a) Proposed reaction pathway and (b) potential energy profile of the amination reaction.

To provide an insight into the mechanism of such reaction, we perform DFT calculation by taking the reaction of **3** and piperidine as an example. All calculations were performed by ORCA 4.2.1 quantum mechanistic software. The transition state geometries were optimized under the BLYP/def2‐SVP level with Grimme's D3 correction. The Gibbs free energy was obtained under the B3LYP‐D3/def2‐TZVP level with SMD enabled.^[9]^ The proposed reaction pathway and energy profile are given in Figure 2. For a typical nucleophilic aromatic substitution, an addition‐elimination mechanism is involved. However, the substitution of cyano groups is always not included in the scope as described in Terrier's book.^[10]^ Indeed, our study reveals that the direct addition of piperidine to **3** needs a high energy barrier of 50.73 kcal/mol, thus difficult for the reaction. However, the reaction may be mediated by a complex formation between one molecule of **3** and two molecules of piperidines. The self‐catalyzation by piperidine occurs in the complex to drive the formation of a zwitterionic intermediate by a nucleophilic attack to the *α*‐carbon atom of the cyano group on pyrazine which was partially positively charged and affected by the electron‐withdrawing pyrazine and cyano units. Then, the deprotonation proceeds with the aid of one‐molecule piperidine followed by leaving the cyano group with a C‐CN bond cleavage to form the product. In this mechanism, the step‐determining step is determined by the formation of a complex, which requires a much lower energy barrier of 19.7 kcal/mol. Such a barrier is rational for a 70°C reaction. Moreover, the base catalyzation occurs in the complex, which is in good accordance with the fact that the excess base may benefit the complex formation to accelerate the reaction. For example, when an excess piperidine (ca. 30 equiv.) is employed, the reaction is accelerated remarkably and can be finished even within 1 h. Finally, as the by‐product of hydrocyanic acid is volatile, flammable, and highly toxic to the body, it is suggestive to conduct the reaction with an excess of amine because it not only plays a positive role in increasing the reaction efficiency but also reliable to neutralize the generated acid.

Thanks to the facile method, 10 compounds, named **Py‐1** to **Py‐10**, are obtained in this work. Their structures as well as those with aryl amine substituents were characterized using ^1^H and ^13^C NMR and high‐resolution mass spectroscopies corresponding to a satisfactory data (Figures S1–S42). These molecules possess a notable D‐A structure with the amine unit and cyano group‐substituted pyrazine unit as strong electronic donor and acceptor, respectively. Moreover, they contain different substituents, providing much possibility to tune their photo‐physical properties. The crystal structures (except for **Py‐9** whose crystal is difficult to grow because it contains a turbulent long alkyl chain) show that they adopt a twisted conformation with the peripheral phenyl rings twisting against the central pyrazine ring.^[11]^ The torsion of cyano and amine groups is relatively small, though the aliphatic chain of cyclamine possess a different conformation (Figure S43).

**FIGURE 3:**
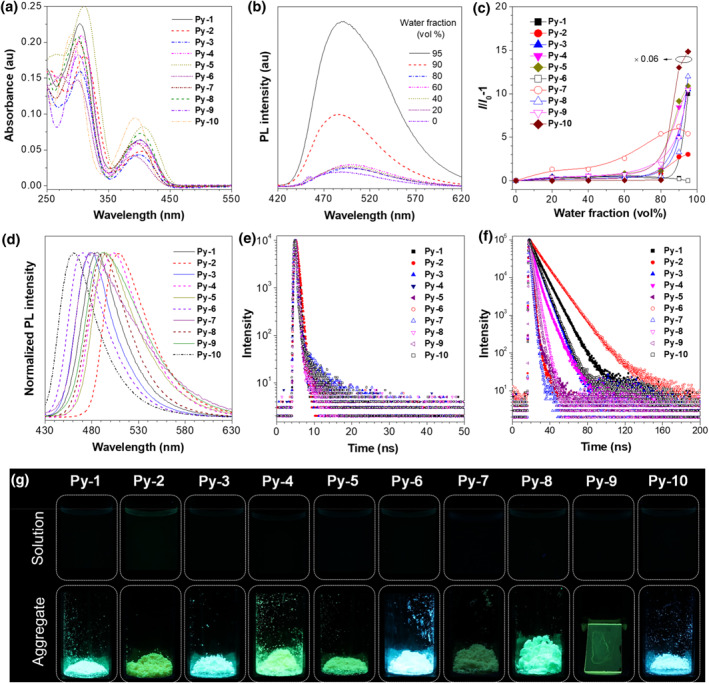
(a) UV‐Vis spectra of AIEgens in THF. (b) PL spectra of **Py‐1** in THF/water mixtures with different *f*_*w*_. (c) Plots of relative PL intensity (*I*/*I*_0_ − 1) of AIEgens versus *f*_*w*_, where *I*_0_ = PL intensity in pure THF solution. (d) PL spectra of AIEgens in the aggregate state. Fluorescent lifetime of AIEgens in the (e) solution and (f) aggregate states. (g) Photographs of (upper) solution and (lower) aggregates of AIEgens taken under 365 nm UV light. The concentration of AIEgen solution is 10 μM. All aggregate‐state samples are tested in powders except for **Py‐9**, which is used as film. AIEgens, aggregation‐induced emission luminogens; PL, photoluminescence.

The UV‐Vis spectra show that **Py‐1** to **Py‐10** have a similar absorption profile, while the peaks at ∼400 and 300 nm are attributable to the charge‐transfer transition from the donors to the acceptors and the local‐state transition of the molecules, respectively (Figure 3a and Table S2). Because the conjugated molecules with twisted conformation are easy to obtain the AIE property according to the principle proposed by Tang,^[12]^ we next examine whether such property could be realized in our system? With this in mind, we studied their photoluminescence (PL) spectra in THF/water mixtures with different water fractions (*f*_*w*_). Take **Py‐1** as an example, it emits weakly in diluted THF. After addition of the water, the emissions change less with the *f*_*w*_ from 0% to 80% but are intensified remarkably afterward (Figure 3b). A similar phenomenon is observable in the molecules except for Py‐6 (Figures 3c and S44). Since THF and water separately act as good and poor solvents, the addition of a large amount of water into THF must impel the molecules to form the aggregates. Thus, the much‐enhanced emission in the THF/water mixture with high *f*_*w*_ than in the solution state is ascribed to AIE. The reason why **Py‐6** shows a relatively weak emission may be attributed to the formation of non‐emissive aggregates.^[13]^ However, the integral sphere test shows that their absolute PL quantum yields (Φ_*F*_) in the aggregate state (e.g., powder or film) range from 3.97% to 70.5%, which are much larger than that in the solution (0.4%–2.60%), confirming that they possess an AIE property (Table S2). Due to the different substituents, the AIEgens behave differently in their AIE effect. Their *α*_AIE_, defined as the ratio of Φ_*F*_ between the aggregate and solution states, change from 3 to 108. Besides, they have wide emissions from deep‐blue to yellow‐green (Figure 3d,g). All these suggest that the aggregate effect and electronic structure may be influenced by the substituents in the AIEgens. Moreover, the transient PL spectra reveal that all the AIEgens have a short excited‐stated lifetime (*τ*) from 0.4 to 12.4 ns, indicating that these emissions belong to a short‐lived fluorescence (Figure 3e,f).

The excited‐state decay rates of these AIEgens are evaluated from the formula of *k*_*r*_ = Φ_*F*_/*τ* and *k*_nr_ = (1 − Φ_*F*_)/*τ*, where *k*_*r*_ and *k*_nr_ are the radiative and nonradiative decay rates, respectively. Their *k*_*r*_ in the solution and aggregate states are in the range of 0.08–0.58 × 10^8^ and 0.27–0.54 × 10^8^ s^−1^, respectively (Table S2). The slight change in *k*_*r*_ implies that the radiative transition from the excited state has been less influenced from the solution to the aggregate states. In a sharp contrast, the *k*_nr_ of AIEgens in the aggregate state are deduced to be 0.24–6.36 × 10^8^ s^−1^, which are much lower than that in the solution (17.4–24.6 × 10^8^ s^−1^). Thus, the suppression of nonradiative transition from excited‐state molecular motion makes a dominant contribution to the AIE behavior. It is particularly evident that the highest Φ_*F*_ is observed in **Py‐6** (70.5%) in the powder because of its remarkable reduction in *k*_nr_ by 95‐fold upon the aggregation (Table S2).

**FIGURE 4:**
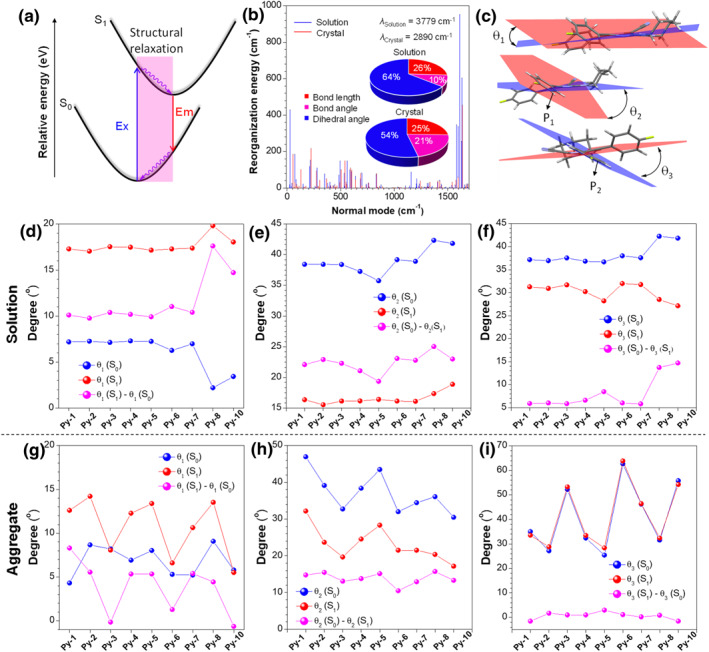
(a) Exhibition of structural relaxation after molecular relaxation in energy level diagram. (b) Calculated reorganization energy of **Py‐1** at different wavenumbers. Inset: projection of reorganization energy on the internal coordination. (c) Indication of changes of (d) and (g) *θ*_1_ (twisted angle of pyrazine ring), (e) and (h) *θ*_2_ (dihedral angle between P_1_ and pyrazine ring) and (f) and (i) *θ*_3_ (dihedral angle between P_2_ and pyrazine ring) of AIEgens in (d), (e), and (f) solution and (g), (h), and (i) solid states. AIEgens, aggregation‐induced emission luminogens.

To give a deeper insight into the AIE process, the reorganization energy (*λ*) of **Py‐1** was investigated using the MOMAP package^[14]^ because it characterizes the excited‐state decay dynamics with detailed information of multiple molecular motions after photo‐excitation, which is regarded as an essential factor to affect the non‐radiative decay process (Figure 4a). The *λ* in the solution and solid state were calculated to be 3779 and 2890 cm^−1^, respectively (Figure 4b). The reduction in *λ* indicates that the nonradiative decay is suppressed due to the restriction of intramolecular motion from the single‐molecule state to the aggregate state, which is in accordance with the experimental results. From the plots of *λ* versus normal model frequencies, the low frequency vibration modes corresponding to the changes of dihedral angle are decreased obviously, while the higher frequency vibration modes associated with the stretching and blending motions of bond change less. Further casting the *λ* onto the internal coordinate indicates that the change in the dihedral angle makes a major contribution (with a proportion of 64% and 54% in the solution state and solid state, respectively) to the total *λ*. The *λ* of this component decreases from 2436 to 1550 cm^−1^ by aggregation, while that from variation in bond length and bond angle poses a minor effect. Thus, the restriction of changes in the dihedral angle plays a significant role in affecting the non‐radiative transition process. However, there are three modules in **Py‐1** which involve such a motion. They are pyrazine, phenyl, and piperidine‐related units, and the *λ* coming from them are deduced as ca. 1365, 760, and 288 cm^−1^ in the solution and 1004, 442, and 52 cm^−1^ in the solid, respectively. It is evident that the pyrazine and phenyl‐related units dominate the molecular motion not only because they possess a large proportion of *λ* (56% and 32% in the solution and 65% and 29% in the solid, respectively) but also have an obvious decrease in *λ* (361 and 318 cm^−1^, respectively) by aggregation.

More intuitive information is extracted from the optimized geometries of **Py‐1** in the solution state and solid state at the energy minimum of their ground state (S_0_) and excited state (S_1_). Actually, the central pyrazine ring in **Py‐1** is naturally twisted and possesses a dihedral angle of *θ*_1_ due to the large dipole induced by the uneven charge distribution caused by the difference in electronegativity between C and N atoms. **Py‐1** has a *θ*_1_ of 7.2° in the S_0_ geometry in the solution. However, the angle increases to 17.3° at the minimum of the S_1_ state (Figure 4c,d). Thus, the pyrazine ring is flapping just like the wings of a flying butterfly in the excited‐state relaxation. On the other hand, two phenyl groups attaching to the pyrazine ring are possible to move in the excited state. The *θ*_2_ and *θ*_3_, defined as the dihedral angles between the pyrazine ring and phenyl rings (P_1_ and P_2_) in **Py‐1**, are 38.4° and 37.2°, respectively, which are larger than that (16.4° and 31.3°) in the S_1_ geometry (Figure 4e,f). Thus, the phenyl group can rotate after light‐excitation. By contrast, the change of *θ*_3_ is much smaller than *θ*_2_ because the electron‐donating amine in the opposite may stabilize the P_2_ by a conjugated or induced effect. Thus, the intramolecular flapping of the pyrazine ring, together with the rotation of phenyl rings, collectively contribute to the non‐radiative transition in the solution state. However, in the solid state, the pyrazine ring is less twisted both in the ground state and excited state, and the dihedral angle variation reduces from 10.1° in the solution to 8.3° in the solid (Figure 4d,g). Besides, the rotation of P_1_ decreased from 22.1° to 14.8° between the solution and solid states (Figure 4e,h). P_2_ shows a slight rotation by 5.9° in the solution, while such motion is negligible in the solid (Figure 4f,i). These results manifest that the suppression of the synergetic motions is an intrinsic cause of the AIE process.

We also performed the structural optimization of other AIEgens and a similar behavior of suppressing molecular motions from the solution to solid states can be observed (Figure 4d–i). However, there are some differences between them. For example, the mobility of pyrazine rings and phenyl rings in **Py‐8** are larger than the other AIEgens in the solution. It corresponds to its highest *k*_nr_ in the solution. Moreover, in the solid state, the motion of the pyrazine ring of **Py‐3**, **Py‐6**, and **Py‐10** is much restricted than the others. Thus, they possess high Φ_*F*_ of 30.7%, 70.5%, and 43%, respectively (Table S2). By contrast, although the flapping of the pyrazine ring in **Py‐6** is somewhat small, the restriction of phenyl rotation is relatively apparent. Both factors collectively contribute to a highest Φ_*F*_. It concludes that both the motions play an important role in the AIE effect, and more motions restricted, higher the luminescent efficiency in the aggregate. It is well known that Tang proposed the AIE in phenyl‐substituted siloes in 2001 and revealed the restriction of intramolecular phenyl ring rotation as the cause.^[15]^ It is also found to explain the AIE behavior of tetraphenylethenes by the restriction of phenyl rotation plus twist of central double bound.^[16]^ Our study reveals that the suppression of intramolecular flapping is indispensable in the AIE effect of compounds obtained from the reaction, therefore providing an enrichment to the AIE mechanism.

**FIGURE 5:**
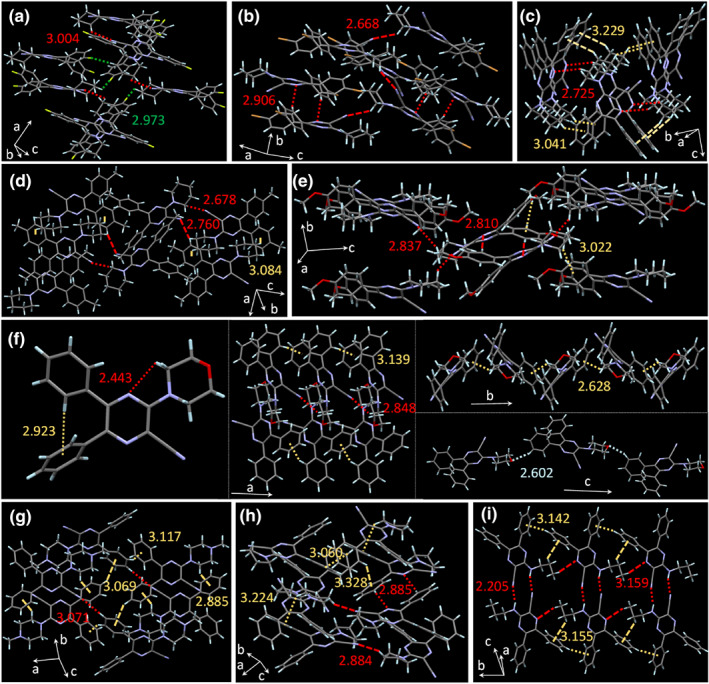
Intermolecular or intramolecular interactions of (a) **Py‐1**, (b) **Py‐2**, (c) **Py‐3**, (d) **Py‐4**, (e) **Py‐5**, (f) **Py‐6**, (g) **Py‐7**, (h) **Py‐8**, and (i) **Py‐10** in crystals with indicated distance (Å). The C‐H···N, C‐H···*π*, C‐H···F and C‐H···O interactions were marked with red, yellow, green and light blue, respectively.

We then investigate the factors that affect their aggregate‐state behavior by extracting the information from the crystal structure. Thanks to their twisted conformation caused by the congested phenyl rings and bulky aliphatic chains, the molecules adopt a non‐face‐to‐face arrangement, while the *π*‐*π* stacking effect is exemptible to quench the fluorescence (Figure 5). Instead, there are plenty of intramolecular (e.g., C‐H···N and C‐H···*π*) and intermolecular (e.g., C‐H···N, C‐H···*π*, C‐H···F, and C‐H···O) interactions, which can help to lock the molecular motions to contribute to the luminescence. However, the substituents have a profound effect on the aggregate‐state behavior as well as the luminescence. In **Py‐2**, **Py‐5**, and **Py‐7**, the presence of bulky bromine atoms, methoxyl groups on the phenyl rings, and methyl groups in the 1‐methylpiperazine group increase the intermolecular gap (Figure 5b,e,g). Despite that some C‐H···N interactions between the H atoms from amines or phenyl rings and N atoms from cyano groups or pyrazine rings with *d* = 2.668–3.071 Å and C‐H···*π* interactions between H atoms from methoxyl group, amines, or benzene groups and phenyl rings with *d* = 2.885–3.022 Å still exist between the molecules, the excess of space enables the molecular motions to occur inevitably. This corresponds to their large *k*_nr_ of 5.05 × 10^8^, 5.65 × 10^8^, and 6.36 × 10^8^ s^−1^ and small Φ_*F*_ of 8.17%, 8.53%, and 3.77% in the aggregate state, respectively (Table S2). By contrast, much‐crowed packing assisted by the rich intermolecular interactions is found in AIEgens with other substituents, which is better for suppressing intramolecular motions. In **Py‐1**, the intermolecular C‐H···F interaction between H atoms of amine and F atoms on benzene rings is formed with *d* = 2.973 Å, which acts as an additional factor to lock the motions (Figure 5a). However, in **Py‐3**, **Py‐4**, and **Py‐8**, more C‐H···N interactions with a short distance of 2.678–2.885 Å and C‐H···*π* interactions with a distance of 3.041–3.328 Å were generated to stabilize the molecular conformations (Figure 5c,d,h). These enable them to possess moderate Φ_*F*_ from 15.4% to 30.7%. In **Py‐6**, the molecules are physically cross‐linked by three‐dimensional intermolecular interactions, which is different from the most AIEgens that adopt a two‐dimensional linkage (Figure 5f). For example, **Py‐6** is connected by C‐H···N interactions between H atoms from morpholine groups and cyano groups with *d* = 2.848 Å and C‐H··· *π* interactions between H atoms from benzene groups and phenyl rings with *d* = 3.139 Å along the *a* axis, and C‐H··· *π* interactions between H atoms from morpholine groups and phenyl rings with *d* = 2.628 Å along the *b* axis, while the O atoms in the morpholine groups provide action sites to bond with the H atoms of phenyl rings in adjacent molecules with a short distance of 2.602 Å along the *c* axis. Besides, **Py‐6** can self‐lock the conformation by forming the C‐H··· *π* interaction between H atoms from benzene rings and adjacent phenyl groups with *d* = 2.923 Å and C‐H···N interaction between H atoms from morpholine groups and N atoms from pyrazine rings with *d* = 2.443 Å, while only the latter interaction can be found in other AIEgens (Figure S45). Thus, the collective internal and external factors enable **Py‐6** to possess the best restriction of intramolecular motions and the highest Φ_*F*_ in the aggregate state. By comparison, **Py‐10** only shows a one‐dimensional connection along the *b* axis but has a high Φ_*F*_ of 43.0% (Figure 5i). This is due to the high hydrogen bond density in the one‐dimensional molecular belt, which creates a favorable condition for the intramolecular motion restriction. For example, an extremely strong intermolecular C‐H···N interaction with *d* = 2.205 Å is formed between molecules head by head. Besides, the propyl group also provides the sufficient hydrogen sites to generate C‐H···N interaction with N atoms of pyrazine rings and C‐H···*π* interaction with phenyl rings in the adjacent molecules.

**FIGURE 6:**
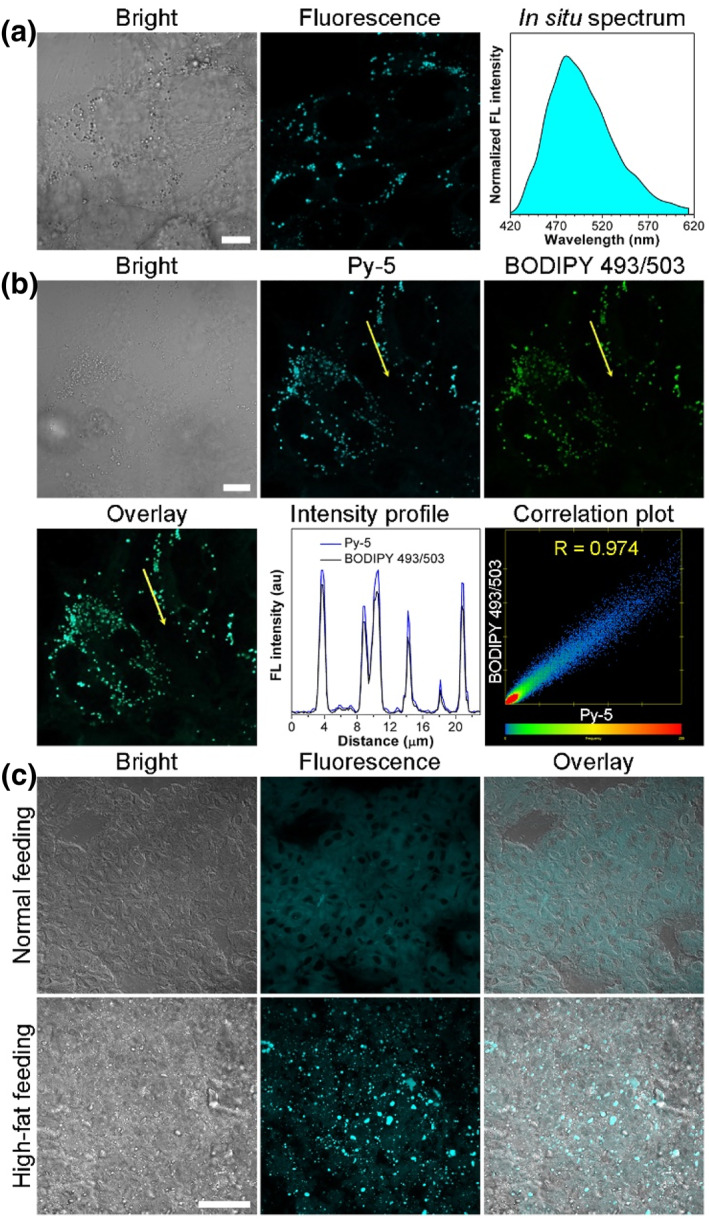
(a) Confocal laser scanning microscopy images of HeLa cells stained with **Py‐5** (1 μM) and its in situ fluorescence spectra. Scale bar: 10 μm. (b) Confocal laser scanning microscopy images of HeLa cells stained with **Py‐5** (1 μM) and BODIPY 493/503 (0.2 μM), the intensity profile within the regions of interest (indicated by yellow arrows), and the fluorescence intensity correlation plot of **Py‐5** and BODIPY 493/503. *R* is the Pearson's coefficient. Scale bar: 10 μm. (c) Confocal laser scanning microscopy images of normal and high‐fat feeding guinea pig liver tissues stained with Py‐5 (4 μM). Scale bar: 50 μm.

Biological fluorescence imaging is endowed with the merits of quick response, high spatial resolution, excellent sensitivity and good selectivity, etc.^[17]^ With the impressive photophysical properties of these AIEgens in hand, we further carried out the fluorescence imaging to study their biological applications. Before bioimaging, we first evaluated the potential cytotoxicity of these AIEgens in live HeLa cells via a standard MTT assay. After incubation with HeLa cells for 24 h, the viability of HeLa cells is still very high (>83%) even at a concentration of 10 μM (Figure S46), indicative of a low cytotoxicity of the AIEgens toward live HeLa cells. Then, we performed live cell imaging by a confocal laser scanning microscopy. After incubated with HeLa cells for 20 min, it clearly shows in Figures 6a and S48 that the bright spherical spots with high fluorescence contrast from **Py‐1** to **Py‐9** can be observed, revealing that they have an excellent membrane permeability. The in situ fluorescence spectra in HeLa cells show that they emit sky‐blue fluorescence (459–479 nm, Figures 6a and S47). Considering the hydrophobic structures of these AIEgens and the inherent lipophilic environment of lipid droplets, we anticipate that these AIEgens are probably located in the lipid droplets according to the theory of similarity and intermiscibility.^[18]^ To confirm the possible selective staining in lipid droplets, the co‐staining experiments with a commercial lipid droplet dye BODIPY 493/503 were conducted (Figures 6b and S48). Except for **Py‐7** with a 4‐methylpiperazine group, other AIEgens show a very similar imaging behavior in HeLa cells to that of BODIPY 493/503, and the corresponding Pearson's coefficients range from 0.972 to 0.986. Obviously, these AIEgens except for **Py‐7** are selectively located in the lipid droplets. The reason why **Py‐7** shows a low selectivity in the lipid droplets probably may be that the 4‐methylpiperazine substituted dyes or probes tend to locate in lysosomes.^[19]^ It should be noted that the commercially available lipid droplet probes, BODIPY 493/503 and Nile Red, are excited under 488 or 543 nm laser.^[18b,18c]^ To avoid a spectral cross‐talk in co‐stain imaging experiments, our biocompatible AIEgens can serve as excellent blue‐emissive lipid droplet probes.

Some previous studies have indicated that fatty liver diseases are characterized by excess lipid storage in lipid droplets accumulating in hepatocytes.^[20]^ Encouraged by the excellent in vitro lipid droplet imaging performance, we further check out whether these D‐A type AIEgens could fluorescently discriminate the fatty liver disease tissues from the normal liver tissues. The fatty liver disease model is built by providing a high‐fat feeding diet to male Hartley guinea pigs, while another group of guinea pigs with a normal feeding diet under the same experimental conditions is set as a control. Using **Py‐5** as an example, we incubated the sectioned liver tissues from guinea pigs with this AIEgen for 0.5 h before confocal fluorescence imaging. As displayed in Figure 6c, **Py‐5** shows a weak fluorescence in the normal feeding guinea pig liver tissues. In contrast, a strong fluorescence of **Py‐5** in spherical spots with a high signal‐to‐noise ratio is collected in the high‐fat feeding guinea pig liver tissues due to the low polar viscous environment in lipid droplets and its intramolecular charge transfer effect. Presently, Oil Red O staining has become a widely used method for fatty liver disease diagnosis by visualization of lipid and fat deposits in liver biopsy samples under a bright‐field microscopy.^[21]^ However, for Oil Red O staining, multiple and complicated procedures for the tissue treatment are required before satisfying images are acquired, which is time‐consuming (see Supporting Information S1). On the other hand, we further conducted Oil Red O staining of high‐fat feeding guinea pig liver tissues at different concentrations. As shown in Figure S49, the lipid droplets could be clearly displayed by Oil Red O at a high concentration of 12 mM. After decreasing the concentration to 1.2 mM, the red color of lipid droplets (marked by green arrows) become distinctly darkened and blurry, let alone the imaging performed at a micromole concentration. In a sharp contrast, our AIEgen **Py‐5** could even work at 4 μM to discriminate the fatty liver disease tissues by lighting‐up the lipid droplets (Figure 6c), indicative of a better sensitivity than Oil Red O staining. Thus, the remarkable fluorescence imaging performances render our AIEgens as potential tools for the diagnosis of fatty liver diseases from a liver biops.

## 3 CONCLUSION

In this work, we report a facile method to realize D‐A AIEgens by a complex‐mediated nucleophilic aromatic substitution of pyrazine‐based aryl nitriles with amines. The reactions can proceed efficiently without transition metal catalyst in THF under heated and air conditions. In comparison to the conventional transition metal‐catalyzed synthesis, our method shows obvious advantages in economy and environmental conservation, and it can facilely access to D‐A compounds used in medical and material areas for a large‐scale production. The resulting AIEgens show a tunable photophysical property affected by the substitutions. They exhibit deep‐blue to yellow‐green emissions and high luminescent efficiencies up to 70.5% in the aggregate state. Interestingly, the suppression of intramolecular flapping is proved to play an indispensable role in the contribution to the AIE behavior, therefore enriching the AIE mechanism. We also confirm that the multiple intramolecular and intermolecular interactions can help to lock the molecular motion in the aggregate state to result in bright emissions. It is noteworthy that increasing the intermolecular interactions by a three‐dimensional connection and local hydrogen bond density are beneficial to prohibit the molecular motions. Moreover, these AIEgens show good biocompatibility and excellent targeting ability towards lipid droplets in HeLa cells. Using **Py‐5** as an example, we successfully demonstrate their capability in discriminating the fatty liver tissue from normal liver tissue by fluorescent imaging at a high contrast. Compared with traditional Oil Red O staining, our method is handy and shows much more excellent sensitivity. Thus, the unique D‐A AIEgens can function as fluorescent probes in visualization of lipid droplet associated biological behavior and fatty liver diseases. Our synthetic method provides great opportunities in preparing various fluorescent materials for advanced applications.

## CONFLICT OF INTEREST STATEMENT

The authors declare no conflicts of interest.

## ETHICS STATEMENT

All animal experiments were approved by the institutional committee and completed at the Experimental Animal Center of Guangzhou University of Chinese Medicine (SYXK (Yue) 2018‐0085).

## Supporting information

Supporting Information S1

## Data Availability

The data that support the findings of this work are available in the Supporting Information S1 of this article.

## References

[1] a) S. Günes , H. Neugebauer , N. S. Sariciftci , Chem. Rev. 2007, 107, 1324; 17428026 10.1021/cr050149z

[2] a) J. Mei , N. L. C. Leung , R. T. K. Kwok , J. W. Y. Lam , B. Z. Tang , Chem. Rev. 2015, 115, 11718; 26492387 10.1021/acs.chemrev.5b00263

[3] a) K. K. C. Chong , B. Liu , Acc. Chem. Res. 2019, 52, 3051; 31588730 10.1021/acs.accounts.9b00356

[4] a) Y. Wang , Z. He , G. Chen , T. Shan , W. Yuan , P. Lu , Y. Zhang , Chin. Chem. Lett. 2017, 28, 2133;

[5] a) G. Yan , Y. Zhang , J. Wang , Adv. Synth. Catal. 2017, 359, 4068; 10.1002/adsc.201700438 PMC5844573 29531521

[6] a) F. F. Fleming , L. Yao , P. C. Ravikumar , L. Funk , B. C. Shook , J. Med. Chem. 2010, 53, 7902; 20804202 10.1021/jm100762r PMC2988972

[7] a) J. Jayakumar , T. L. Wu , M. J. Huang , P. Y. Huang , T. Y. Chou , C. H. Cheng , ACS Appl. Mater. Interfaces 2019, 11, 21042; 31088068 10.1021/acsami.9b04664

[8] a) W. D. Rounds , J. T. Eaton , J. H. Urbanowicz , G. W. Gribble , Tetrahedron Lett. 1988, 29, 6557;

[9] a) F. Neese , WIREs Comput. Mol. Sci. 2012, 2, 73;

[10] F. Terrier , Modern Nucleophilic Aromatic Substitution, Wiley, Weinheim 2013.

[11] Deposition numbers: 2074168 (Py‐1), 2074169 (Py‐2), 2074170 (Py‐3), 2074171 (Py‐4), 2074174 (Py‐5), 2074175 (Py‐6), 2074180 (Py‐7), 2074181 (Py‐8), and 2074182 (Py‐10).

[12] a) J. Mei , Y. Hong , J. W. Y. Lam , A. Qin , Y. Tang , B. Z. Tang , Adv. Mater. 2014, 20, 5429; 10.1002/adma.201401356 24975272

[13] a) X. Luo , J. Li , C. Li , L. Heng , Y. Q. Dong , Z. Liu , Z. Bo , B. Z. Tang , Adv. Mater. 2011, 23, 3261; 21678499 10.1002/adma.201101059

[14] a) Z. Shuai , Chin. J. Chem. 2020, 38, 1223;

[15] J. Chen , C. C. W. Law , J. W. Y. Lam , Y. Dong , S. M. F. Lo , I. D. Williams , D. Zhu , B. Z. Tang , Chem. Mater. 2003, 15, 1535.

[16] a) J. B. Xiong , Y. X. Yuan , L. Wang , J. P. Sun , W. G. Qiao , H. C. Zhang , M. Duan , H. Han , S. Zhang , Y. S. Zheng , Org. Lett. 2018, 20, 373; 29303592 10.1021/acs.orglett.7b03662

[17] a) M. Yang , Z. Özdemir , H. Kim , S. Nah , E. Abdris , X. Li , Z. Wimmer , J. Yoon , Adv. Healthcare Mater. 2022, 11, 2200529; 10.1002/adhm.202200529 35536751

[18] a) G. Niu , R. Zhang , J. P. C. Kwong , J. W. Y. Lam , C. Chen , J. Wang , Y. Chen , X. Feng , R. T. K. Kwok , H. H. Y. Sung , I. D. Williams , M. R. J. Elsegood , J. Qu , C. Ma , K. S. Wong , X. Yu , B. Z. Tang , Chem. Mater. 2018, 30, 4778;

[19] X. Shi , N. Yan , G. Niu , S. H. P. Sung , Z. Liu , J. Liu , R. T. K. Kwok , J. W. Y. Lam , W. X. Wang , H. H. Y. Sung , I. D. Williams , B. Z. Tang , Chem. Sci. 2020, 11, 3152. 34122820 10.1039/c9sc06226b PMC8157324

[20] a) T. K. F. Fam , A. S. Klymchenko , M. Collot , Materials 2018, 11, 1768; 30231571 10.3390/ma11091768 PMC6163203

[21] A. Mehlem , C. E. Hagberg , L. Muhl , U. Eriksson , A. Falkevall , Nat. Protoc. 2013, 8, 1149. 23702831 10.1038/nprot.2013.055

