# Supplementary material for: Complex‐mediated nucleophilic aromatic substitution with aryl nitriles to realize intramolecular flapping‐restricted D‐A AIEgens for bioimaging
Source: Smart Mol. 2024 Nov 14;3(2):e20240039. doi: 10.1002/smo.20240039 (PMC12262011; doi:10.1002/smo.20240039)
Supplement: Supplementary file 1 — Supporting Information S1 [file SMO2-3-e20240039-s001.pdf]

Supporting Information

Complex-mediated nucleophilic aromatic substitution with aryl nitriles to realize intramolecular flapping-restricted D-A AIEgens for bioimaging

Feng Liu,‡[1] Junkai Liu,‡[2] Junyi Gong,‡[3] Runfeng Lin,[1] Siyuan Qiu,[1] Zicheng Liu,[1] Chongyang Li,[1] Miao Meng,[1] Shijie Li,[4] Mei Tu,[1] Jacky W. Y. Lam,[2] Guangle Niu,*[5] Ming Chen*[1]

[1] College of Chemistry and Materials Science, Jinan University, Guangzhou 510632, China

[2] Department of Chemistry, Hong Kong Branch of Chinese National Engineering Research Center for Tissue Restoration and Reconstruction, Department of Chemical and Biological Engineering, The Hong Kong University of Science and Technology, Clear Water Bay, Kowloon, Hong Kong, China

[3] School of Science and Engineering, Shenzhen Institute of Aggregate Science and Technology, The Chinese University of Hong Kong, Shenzhen (CUHK-SZ), Guangdong 518172, China

[4] Clinical Medical College of Acupuncture Moxibustion and Rehabilitation, Guangzhou University of Chinese Medicine, Guangzhou 510006, China

[5] School of Chemistry and Chemical Engineering, Beijing Institute of Technology, Beijing 100081, China

‡ These authors contributed equally to this work.

E-mail: niugl@bit.edu.cn; chenming@jnu.edu.cn

Experimental Section

Materials and Instrumentation: All chemicals were purchased from J&K and Energy Chemical, etc., and used directly without further purification. 2,3-dicyano-5,6-diphenylpyrazine (**3**) is prepared according to the previous literature.[1] ^{1}H and ^{13}C spectra were performed on a Bruker AVIII 400 MHz NMR spectrometer with $CDCl_3$/CD_2Cl_2 as the solvent. High-resolution mass spectra (HRMS) were recorded on a GCT premier CAB048 mass spectrometer in MALDI-TOF mode. UV-Vis spectra were carried out on a Shimadzu UV-2600 spectrophotometer. PL spectra were performed on a Horiba Fluoromax-4 spectrofluorometer. The absolute qutantum yields (Φ_F) were recorded using a Hamamatsu Quantaurus-QY C11347 spectrometer. The PL decay curves were recorded on a Hamamatsu C11367-11 Quantaurus-Yau time-resolved spectrometer. The single crystal X-ray diffractions were carried out with a Rigaku XtaLAB PRO diffractometer.

Synthesis of 2,3-dicyano-5,6-diphenylpyrazine-based derivatives

General procedure: Into a 100 mL round bottom flask was added 5 mmol of benzil-based derivative, 5.5 mmol of daminomaleonitrile in 50 mL acetic acid. The mixture was stirred at reflux for 8 h. After cooled to room temperature, the mixture was poured into a large amount of ice water and extracted by dichloromethane. The organic phase was washed by water several times and then dried over anhydrous Na_2SO_4. The filtrate was concentrated and crude product was purified by a silica gel column with dichloromethane/petroleum ether (v/v = 1:2) as eluent.

Compound **1**: A white powder of 0.986 g was obtained in a yield of 62%. ^{1}H NMR (400 MHz, $CDCl_3$), δ (ppm): 7.61–7.75 (m, 4H), 7.14 (t, J = 8.0 Hz, 4H). ^{13}C NMR (100 MHz, $CDCl_3$), δ (ppm): 165.8, 163.3, 154.1, 132.2, 131.3, 131.2, 129.9, 116.6, 116. 4, 113.1.

Compound **2**: A white powder of 1.76 g was obtained in a yield of 80%. ^{1}H NMR (400 MHz, $CDCl_3$), δ (ppm): 7.59 (d, J = 12.0 Hz, 4H), 7.47 (d, J = 12.0 Hz, 4H). ^{13}C NMR (100 MHz, $CDCl_3$), δ (ppm): 154.1, 133.8, 132.5, 131.4, 130.1, 126.7, 113.0.

Compound **4**: A white powder of 1.43 g was obtained in a yield of 92%. ^{1}H NMR (400 MHz, $CDCl_3$), δ (ppm): 7.49 (d, J = 8.0 Hz, 4H), 7.21 (d, J = 8.0 Hz, 4H), 2.42 (s, 6H). ^{13}C NMR (100 MHz, $CDCl_3$), δ (ppm): 155.3, 142.0, 132.7, 129.9, 129.7, 129.5, 113.5, 21.7.

Compound **5**: A yellow-green powder of 1.20 g was obtained in a yield of 70%. ^{1}H NMR (400 MHz, $CDCl_3$), δ (ppm): 7.59 (d, J = 12.0 Hz, 4H), 6.91 (d, J = 8.0 Hz, 4H), 3.87 (s, 6H). ^{13}C NMR (100 MHz, $CDCl_3$), δ (ppm): 162.0, 154.3, 131.5, 128.7, 127.7, 114.3, 113.4, 55.5.

Synthesis of donor-acceptor type AIEgens with aliphatic amines

General procedure: Into a 25 mL of round bottom flask was added 2,3-dicyano-5,6-diphenylpyrazine-based derivatives (100 mg, *ca.* 0.35 mmol), amines and 1 mL of tetrahydrofuran. The mixture was stirred at 70 °C until the reaction was finished by TLC monitor. The mixture was concentrated and crude product was purified by a silica gel column with dichloromethane/ petroleum ether (v/v = 1:1) as eluent.

Py-1: A yellow-green powder of 122.4 mg was obtained in a yield of 93%. ^{1}H NMR (400 MHz, $CDCl_3$), δ (ppm): 7.51–7.47 (m, 2H), 7.43–7.39 (m, 2H), 7.04–7.01 (m, 4H), 3.90 (s, 4H), 1.78 (s, 6H). ^{13}C NMR (100 MHz, $CDCl_3$), δ (ppm): 164.9, 164.1, 162.4, 161.6, 153.9, 150.7, 141.5, 133.7, 133.4, 132.1, 132.0, 131.1, 131.0, 117.7, 115.7, 115.5, 109.9, 48.5, 26.0, 24.6. HRMS (MALDI-TOF): *m/z* 376.1517 ([M]$^+$), calcd for $C_{22}H_{18}F_2N_4$ 376.1500.

Py-2: An orange powder of 150.3 mg was obtained in yield of 86.6%. ^{1}H NMR (400 MHz, $CDCl_3$), δ (ppm): 7.47–7.42 (m, 4H), 7.36–7.26 (m, 4H), 3.88 (s, 4H), 1.76 (s, 6H). ^{13}C NMR (100 MHz, $CDCl_3$), δ (ppm): 153.8, 150.7, 141.3, 136.5, 136.1, 131.8, 131.6, 130.9, 124.6, 123.0, 117.6, 110.1, 48.5, 26.1, 24.6. HRMS (MALDI-TOF): *m/z* 495.9907 ([M]$^+$), calcd for $C_{22}H_{18}Br_2N_4$ 495.9898.

Py-3: A yellow-green powder of 108.5 mg was obtained in a yield of 91.2%. ^{1}H NMR (400 MHz, $CDCl_3$), δ (ppm): 7.52 (d, J = 8.0 Hz, 2H), 7.44 (d, J = 4.0 Hz, 2H), 7.38–7.28 (m, 6H), 3.90 (s, 4H), 1.78 (s, 6H). ^{13}C NMR (100 MHz, $CDCl_3$), δ (ppm): 154.1, 152.0, 142.9, 137.9, 137.5, 130.0, 129.7, 129.3, 128.4, 128.3, 117.9, 109.9, 48.5, 26.0, 24.6. HRMS (MALDI-TOF): *m/z* 340.1675 ([M]$^+$), calcd for $C_{22}H_{20}N_4$ 340.1688.

Py-4: A yellow powder of 97.6 mg was obtained in a yield of 75.8%. ^{1}H NMR (400 MHz, $CDCl_3$), δ (ppm): 7.41 (d, J = 8.0 Hz, 2H), 7.33 (d, J = 8.0 Hz, 2H), 7.11–7.08 (m, 4H), 3.86 (s, 4H), 2.35 (d, J = 4.0 Hz, 6H), 1.75 (s, 6H). ^{13}C NMR (100 MHz, $CDCl_3$), δ (ppm): 154.1, 151.8, 143.0, 139.9, 138.2, 135.2, 134.8, 130.0, 129.2, 129.1, 118.0, 109.6, 48.5, 26.1, 24.7, 21.6, 21.5. HRMS (MALDI-TOF): *m/z* 368.2001 ([M]$^+$), calcd for $C_{24}H_{24}N_4$ 368.2001.

Py-5: A yellow powder of 117.9 mg was obtained in a yield of 84.2%. ^{1}H NMR (400 MHz, $CDCl_3$), δ (ppm): 7.51 (d, J = 8.0 Hz, 2H), 7.41 (d, J = 8.0 Hz, 2H), 6.86–6.83 (m, 4H), 3.87 (s, 4H), 3.84–3.83 (m, 6H), 1.77 (s, 6H). ^{13}C NMR (100 MHz, $CDCl_3$), δ (ppm): 160.8, 159.7, 154.0, 151.1, 142.5, 132.5, 130.5, 130.3, 118.1, 114.4, 113.9, 113.7, 109.2, 55.4, 48.5, 26.0, 24.6. HRMS (MALDI-TOF): *m/z* 400.1895 ([M]$^+$), calcd for $C_{24}H_{24}N_4O_2$ 400.1899.

Py-6: A pale green powder of 119.5 mg was obtained in a yield of 99.8%. ^{1}H NMR (400 MHz, $CDCl_3$), δ (ppm): 7.49–7.47 (m, 2H), 7.43–7.41 (m, 2H), 7.30–7.26 (m, 6H), 3.91–3.90 (m, 8H). ^{13}C NMR (100 MHz, $CDCl_3$), δ (ppm): 154.1, 152.2, 144.0, 137.5, 137.1, 129.9, 129.8, 129.3, 128.6, 128.4, 128.3, 117.5, 110.9, 66.7, 47.5. HRMS (MALDI-TOF): *m/z* 342.1465 ($[M]^+$), calcd for $C_{21}H_{18}N_4O$ 342.1481.

Py-7: A faint yellow powder of 103.7 mg was obtained in a yield of 83.5%. ^{1}H NMR (400 MHz, $CDCl_3$), δ (ppm): 7.49–7.47 (m, 2H), 7.43–7.41 (m, 2H), 7.37–7.28 (m, 6H), 4.00 (t, J = 6.0 Hz, 4H), 2.69 (t, J = 8.0 Hz, 4H), 2.43 (s, 3H). ^{13}C NMR (100 MHz, $CDCl_3$), δ (ppm): 153.9, 152.1, 143.6, 137.6, 137.3, 130.0, 129.8, 129.3, 128.5, 128.4, 128.3, 117.6, 110.6, 54.9, 47.0, 46.2. HRMS (MALDI-TOF): *m/z* 355.1803 ($[M]^+$), calcd for $C_{22}H_{21}N_5$ 355.1797.

Py-8: A faint yellow powder of 106.2 mg was obtained in a yield of 93.1%. ^{1}H NMR (400 MHz, $CDCl_3$), δ (ppm): 7.51–7.49 (m. 2H), 7.41–7.39 (m, 2H), 7.37–7.29 (m, 6H), 3.92–3.89 (t, J = 6.0 Hz, 4H), 2.10–2.07 (m, 4H). ^{13}C NMR (100 MHz, $CDCl_3$), δ (ppm): 153.1, 151.5, 141.9, 138.1, 137.8, 130.0, 129.6, 129.4, 128.4, 128.3, 128.1, 118.6, 107.8, 48.8, 25.7. HRMS (MALDI-TOF): *m/z* 326.1537 ($[M]^+$), calcd for $C_{21}H_{18}N_4$ 326.1531.

Py-9: A faint yellow viscous liquid of 25.6 mg was obtained in a yield of 18.4%. ^{1}H NMR (400 MHz, $CDCl_3$), δ (ppm): 7.49–7.47 (m, 2H), 7.42–7.40 (m, 2H), 7.36–7.28 (m, 6H), 3.80–3.77 (t, J = 6.0 Hz, 2H), 3.39 (s, 3H), 1.78–1.71 (m, 2H), 1.37–1.26 (m, 10H), 0.90–0.86 (t, J = 8.0 Hz, 3H). ^{13}C NMR (100 MHz, $CDCl_3$), δ (ppm): 152.9, 152.2, 142.1, 137.9, 137.6, 130.0, 129.6, 129.3, 128.4, 128.3, 128.2, 118.5, 107.3, 51.7, 38.2, 32.0, 29.6, 29.4, 27.8, 26.9, 22.8, 14.3. HRMS (MALDI-TOF): *m/z* 398.2458 ($[M]^+$), calcd for $C_{26}H_{30}N_4$ 398.2470.

Py-10: A pale green powder of 44.4 mg was obtained in a yield of 40.4%. ^{1}H NMR (400 MHz, $CDCl_3$), δ (ppm): 7.47–7.45 (m, 2H), 7.37–7.27 (m, 8H), 5.35–5.33 (m, 1H), 3.62–3.57 (m, 2H), 1.79–1.69 (m, 2H), 1.06–1.02 (m, 3H). ^{13}C NMR (100 MHz, $CDCl_3$), δ (ppm): 153.8, 153.7, 142.3, 137.9, 137.8, 129.9, 129.5, 129.3, 128.3, 128.1, 128, 115.8, 110.8, 43.1, 22.8, 11.5. HRMS (MALDI-TOF): *m/z* 315.1597 ($[M+H]^+$), calcd for $C_{20}H_{18}N_4$ 314.1531.

Synthesis of donor-acceptor type AIEgens with aryl amines

General procedure: Into a 250 mL round bottom flask was added aryl amine (0.85 mmol) and 10 mL of dry THF under nitrogen. The mixture was cooled down to -78 °C and then 1 mL (2.55 mmol) of n-BuLi was injected slowly. The reaction was stirred at this temperature for 1 h. Afterwards, 200 mg (0.71 mmol) of **3** dissolved in dry THF was added and the reaction was maintained at -

78 °C for 1h. Then, the reaction was restored to room temperature and heat to reflux for 24 h. The NH_4Cl aqueous solution was added to quench the reaction. The mixture was extracted by dichloromethane and the collected organic phase was washed by water three times. The organic phase was concentrated under reduced pressure and the crude product was purified by a silica gel column with dichloromethane/petroleum ether (v/v = 1:5) as eluent.

Py-DPA: A light yellow powder of 264.3 mg was obtained in a yield of 87.8%. ^1H NMR (400 MHz, CD_2Cl_2), δ (ppm): 7.48–7.44 (m, 7H), 7.39–7.24 (m, 13H). ^{13}C NMR (100 MHz, $CDCl_3$): 153.6, 152.6, 145.9, 144.7, 137.2, 136.8, 130.0, 129.8, 129.7, 129.4, 128.8, 128.4, 128.2, 126.3, 126.2, 116.2, 114.8. HRMS (MALDI-TOF): *m*/*z* 447.1586 ($[M+Na]^+$), calcd for $C_{29}H_{20}N_4$ 424.1688.

Py-Cz: A pale yellow powder of 110.6 mg was obtained in a yield of 36.9% after further crystallization. ^1H NMR (400 MHz, $CDCl_3$), δ (ppm): 8.19–8.17 (d, J = 8.0 Hz, 2H), 7.70–7.65 (m, 6H), 7.53–7.36 (m, 10H). ^{13}C NMR (100 MHz, $CDCl_3$): 154.1, 150.9, 146.6, 139.1, 136.4, 136.2, 130.6, 130.1, 129.9, 129.7, 128.8, 128.7, 126.5, 125.1, 122.3, 121.4, 120.6, 114.9, 111.5. HRMS (MALDI-TOF): *m*/*z* 423.1628 ($[M+H]^+$), calcd for $C_{29}H_{18}N_4$ 422.1531.

Details of calculation

Reaction mechanism investigation: All calculations were performed by ORCA 4.2.1 quantum mechanistic suite. The transition state geometry was optimized under BLYP/def2-SVP level with Grimme's D3 correction. The Gibbs free energy was calculated under B3LYP-D3/def2-TZVP level with SMD enabled. [2-5]

Photo-physical investigation: The ground-state (S_0) and excited-state (S_1) geometries of all the pyrazine derivatives in the solution were optimized using Gaussian 16 package[6] at (TD)-M062X/6-311G(d,p) level. The polarizable continuum model (PCM) was used to simulate the THF solution. The electronic structures of all the pyrazine derivatives in the crystal were evaluated with the combined quantum mechanics and molecular mechanics ((QM/MM) method using the ONIOM model based on the single crystal in Gaussian 16 package. We constructed each ONIOM model by cutting a cluster from the single crystal, in which the central molecule was treated as the QM part at (TD)-M062X/6-311G(d,p) level and the surrounding ones acted as the MM part with the universal force field (UFF). The analytical frequencies of optimized S_0 and S_1 conformations of **Py-1** in both the solution and crystal were performed for the further calculation of vibrational

information. Then the electron-vibration analysis was performed for **Py-1** in the MOMAP package.[7-9]

Cell culture and imaging

HeLa cells were cultured in confocal dishes in the culture medium (Dulbecco's modified Eagle medium (DMEM), supplemented with 10% fetal bovine serum and 1% penicillin and streptomycin) in 5% CO_2/air at 37 °C in a humidified incubator for 24 h. Fresh DMEM medium containing 1 μM of **Py-1** to **Py-9** (Stock solution in DMSO, 1 mM) was added to the culture medium of HeLa cells and incubated at 37 °C in 5% CO_2 for 20 min before imaging. For co-staining experiments, HeLa cells were further incubated with 0.2 μM of BODIPY 493/503. The cells were washed with PBS (pH = 7.4) before imaging. The fluorescence imaging was obtained on a Zeiss LSM 800 Confocal Laser Scanning Microscope (For our AIEgens, excitation 405 nm, emission collection 420−500 nm; For BODIPY 493/503, excitation 488 nm, emission collection 500−620 nm).

Animal preparation

All animal experiments were approved by the institutional committee and completed at the Experimental Animal Center of Guangzhou University of Chinese Medicine (SYXK (Yue) 2018-0085).

Liver tissue imaging

The liver tissues were obtained from the groups of guinea pigs with a normal feeding diet and a high-fat feeding diet (For building the fatty liver disease model) at the same conditions.[10] The 10 μm-thickness liver tissues were stained with the culture medium containing **Py-5** (4 μM) in 5% CO_2/air humidified incubator at 37 °C for 0.5 h. Before imaging, the tissues were washed three times with PBS (pH = 7.4). The same experimental condition was used as the live cell imaging.

Oil Red O staining

The Oil Red O staining method refers to Liang's report[11] with a slight modification. The high-fat feeding guinea pig liver tissue slides were air-dried at room temperature for 20 min, then rehydrated, and stained with Oil Red O (0.5%, m/v, dissolved in isopropanol) at different concentrations for another 20 min. The slides were carefully rinsed with 60% isopropanol for 1 min, then counterstained with hematoxylin for 50 s, and rinsed in tap water for 2 min. Finally, the stained tissue slices were photographed by light microscope using Olympus BX-53 Biomicroscope (Olympus Corporation, Japan).

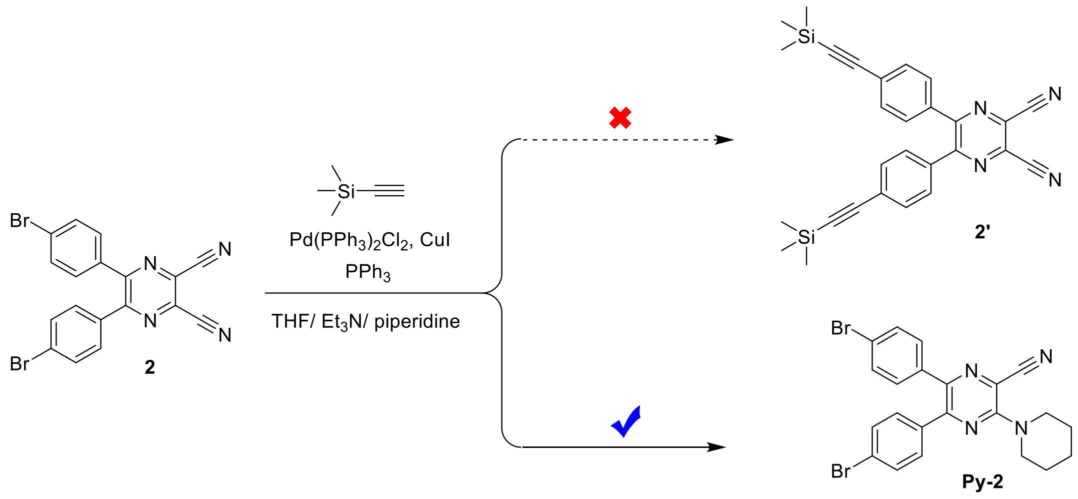

Scheme S1. Unexpected generation of **Py-2** in the Pd-catalyzed Sonogashira reaction with **2**.

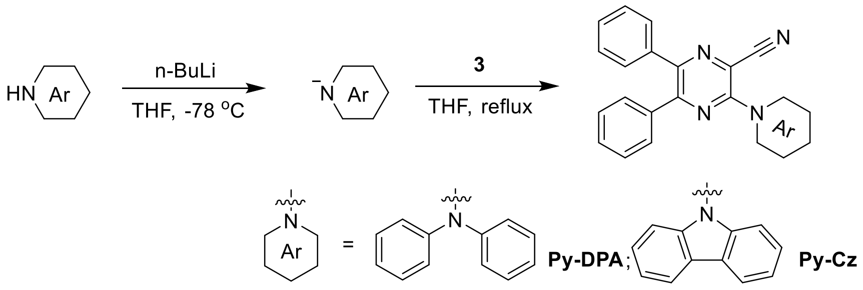

Scheme S2. Synthetic route to compounds **Py-DPA** and **Py-Cz**.

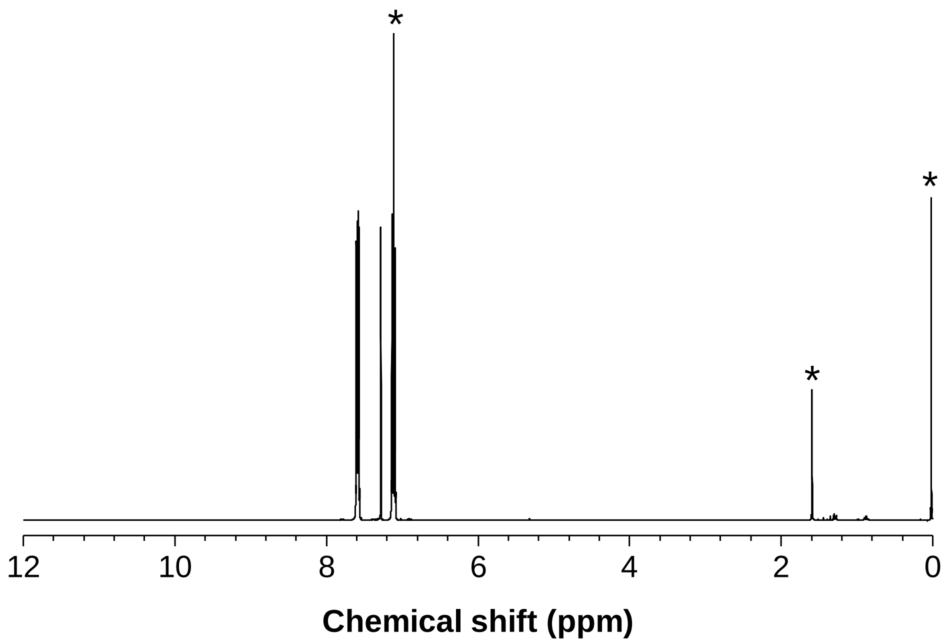

Figure S1. ^{1}H NMR spectrum of **1** in $CDCl_3$. The solvent peaks are marked with asterisks.

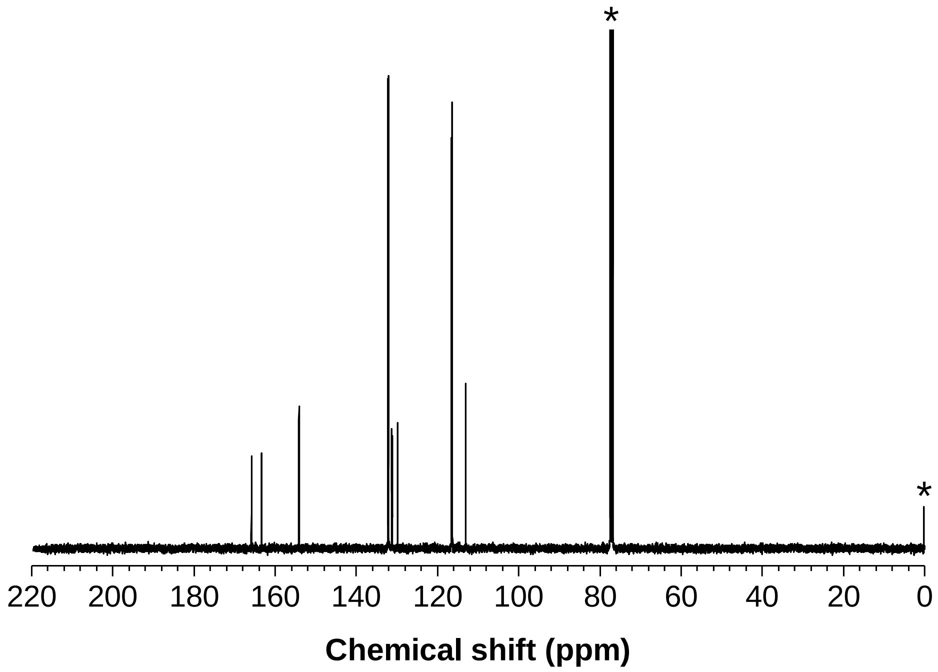

Figure S2. ^{13}C NMR spectrum of **1** in $CDCl_3$. The solvent peaks are marked with asterisks.

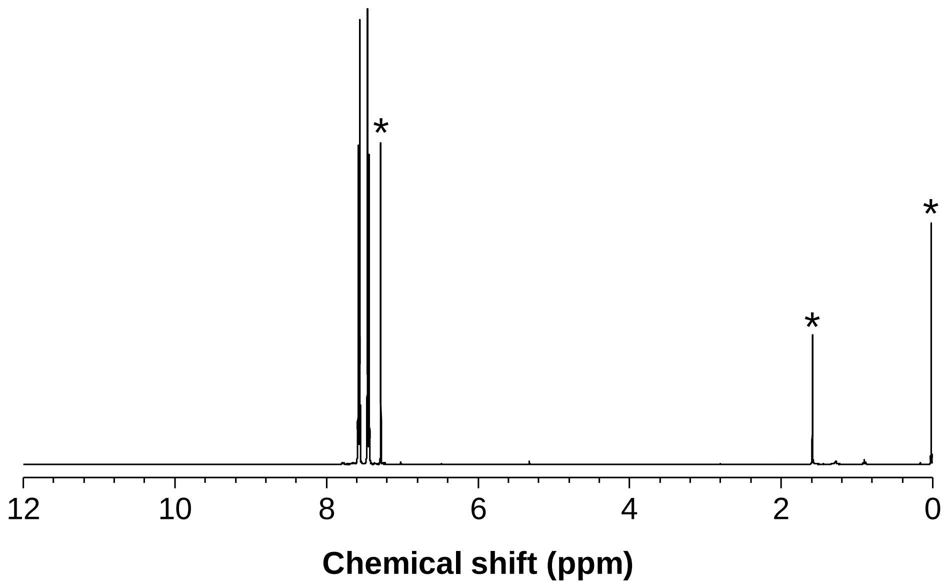

Figure S3. ^{1}H NMR spectrum of **2** in $CDCl_3$. The solvent peaks are marked with asterisks.

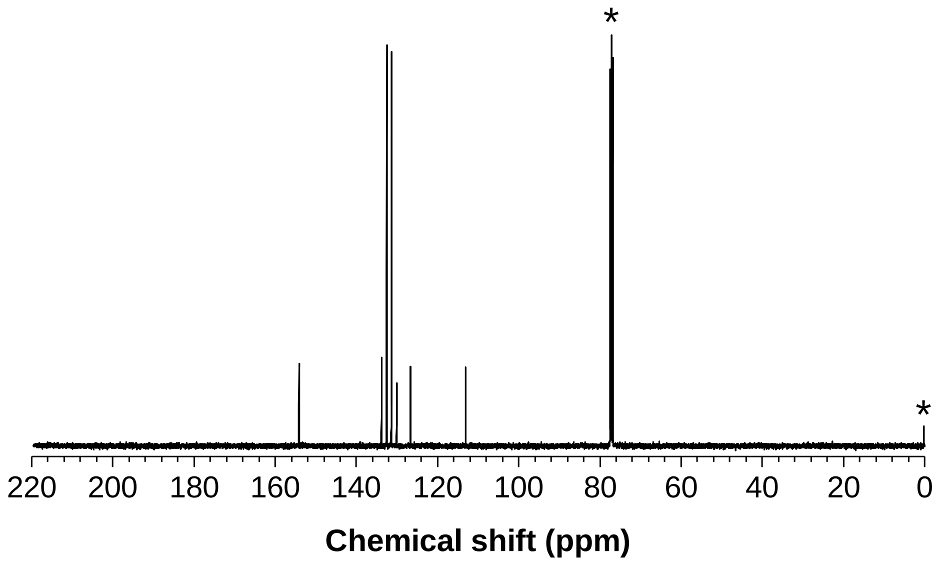

Figure S4. ^{13}C NMR spectrum of **2** in $CDCl_3$. The solvent peaks are marked with asterisks.

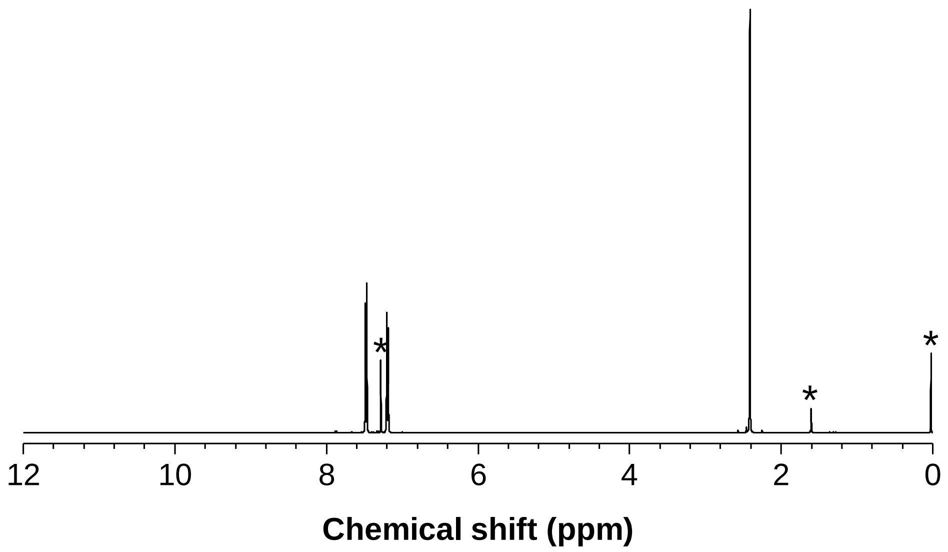

Figure S5. ^{1}H NMR spectrum of **4** in $CDCl_3$. The solvent peaks are marked with asterisks.

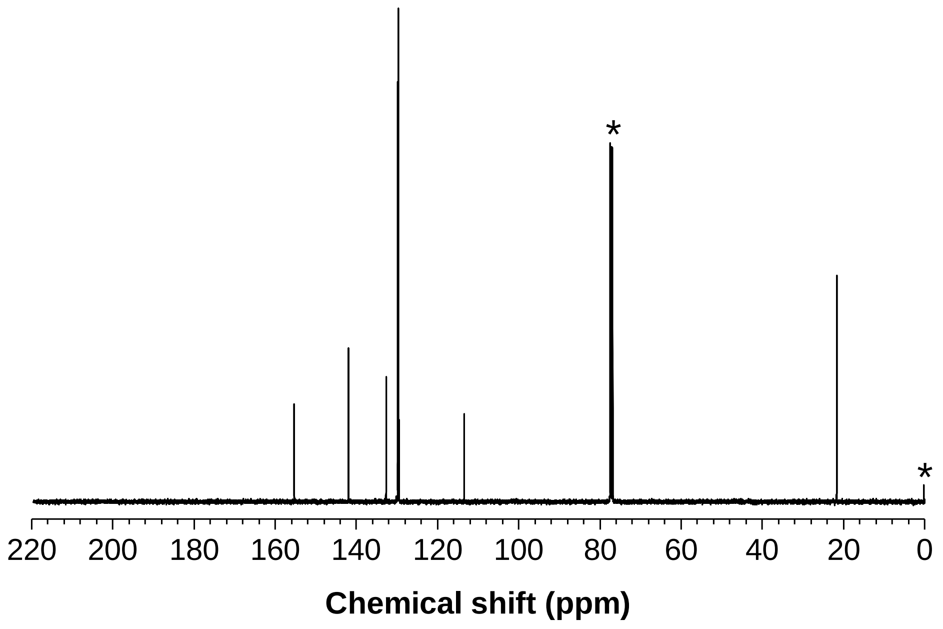

Figure S6. ^{13}C NMR spectrum of **4** in $CDCl_3$. The solvent peaks are marked with asterisks.

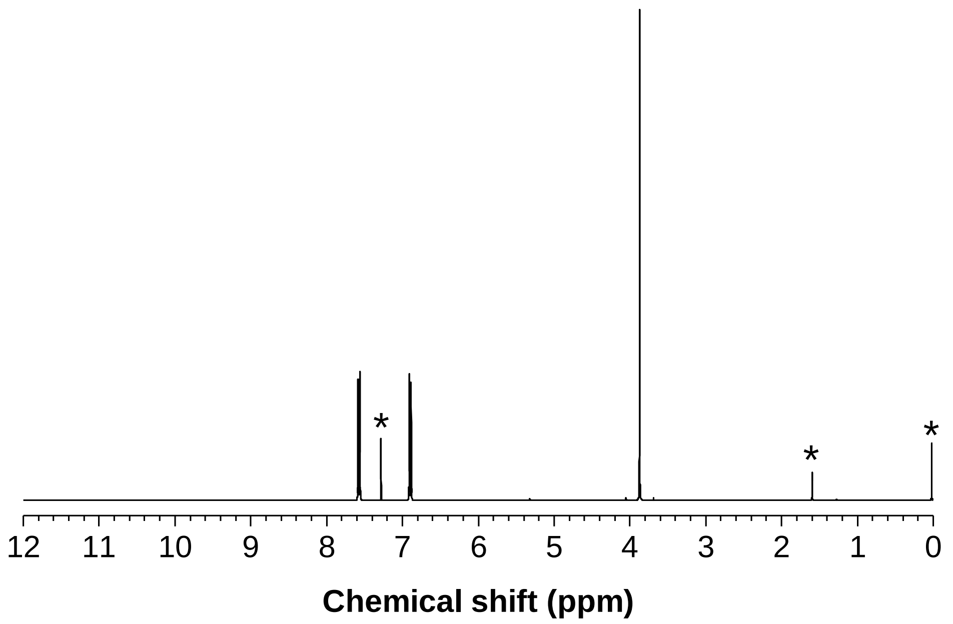

Figure S7. ^{1}H NMR spectrum of **5** in $CDCl_3$. The solvent peaks are marked with asterisks.

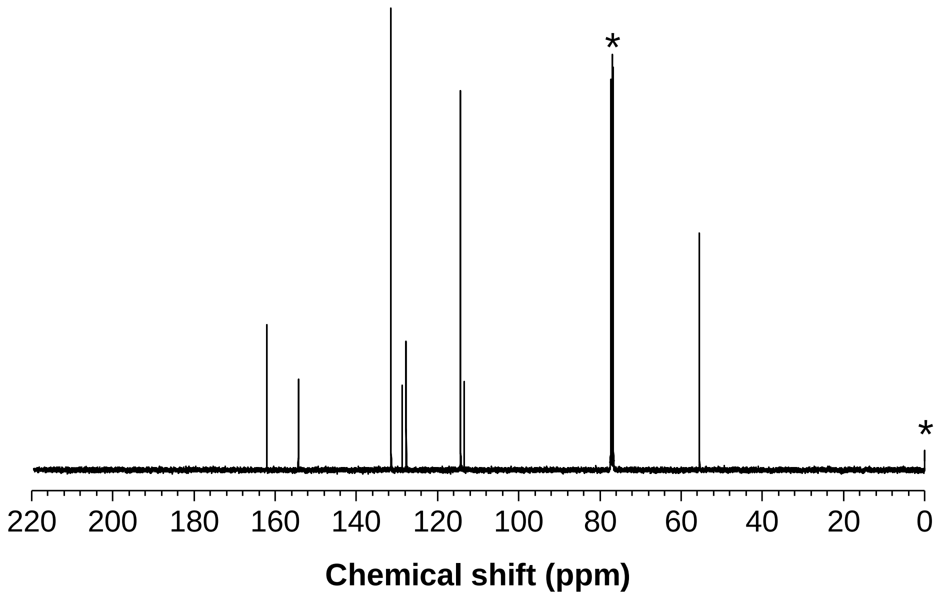

Figure S8. ^{13}C NMR spectrum of **5** in $CDCl_3$. The solvent peaks are marked with asterisks.

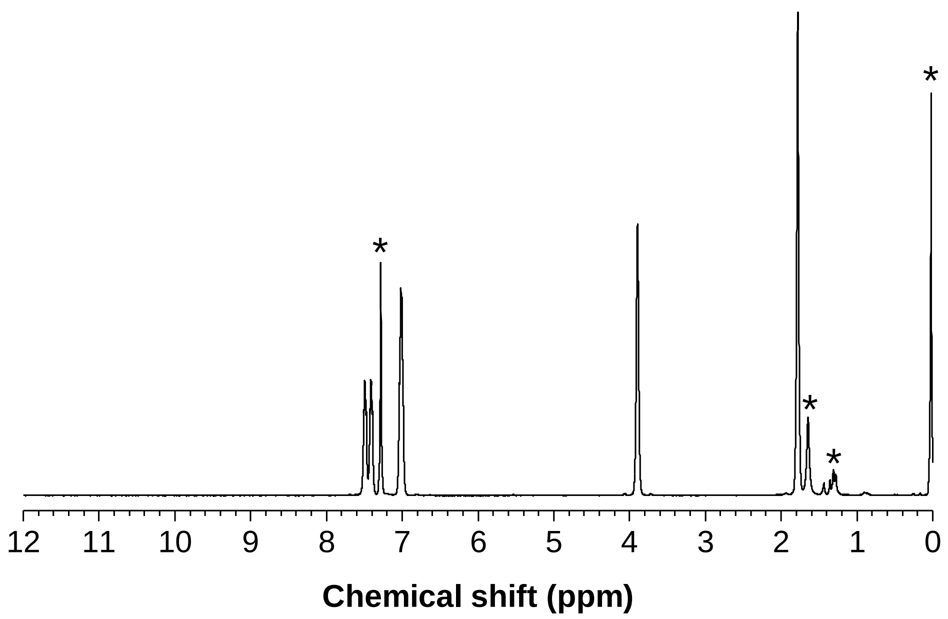

Figure S9. ^{1}H NMR spectrum of **Py-1** in $CDCl_3$. The solvent peaks are marked with asterisks.

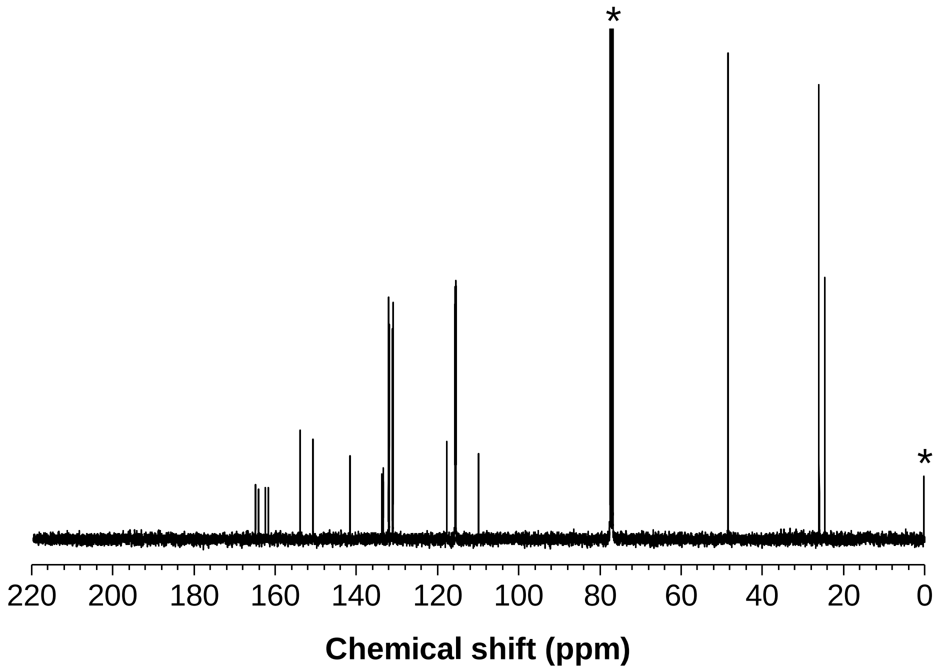

Figure S10. ^{13}C NMR spectrum of **Py-1** in $CDCl_3$. The solvent peaks are marked with asterisks.

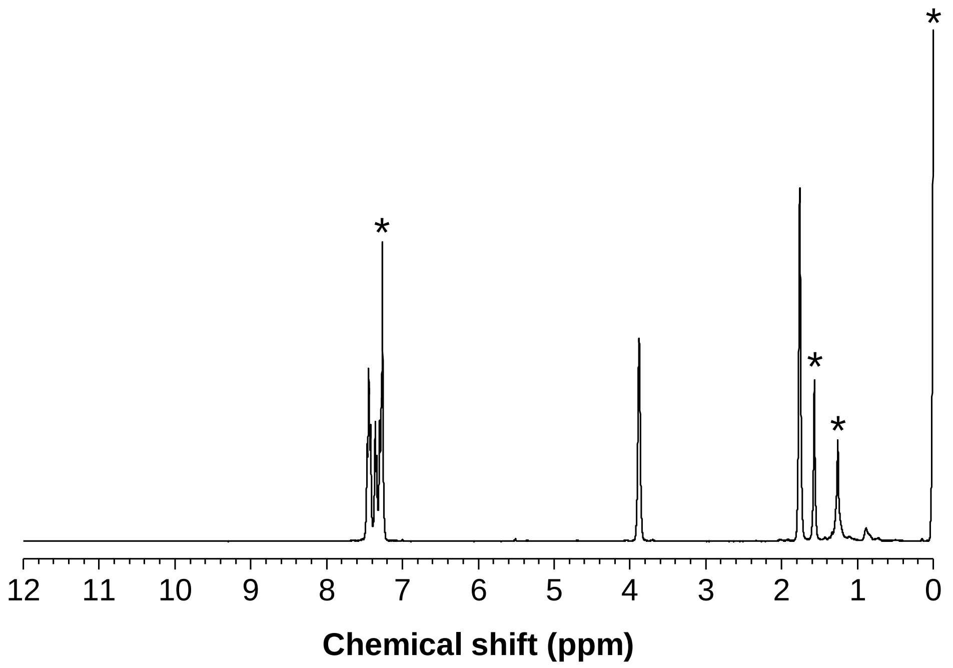

Figure S11. ^{1}H NMR spectrum of **Py-2** in $CDCl_3$. The solvent peaks are marked with asterisks.

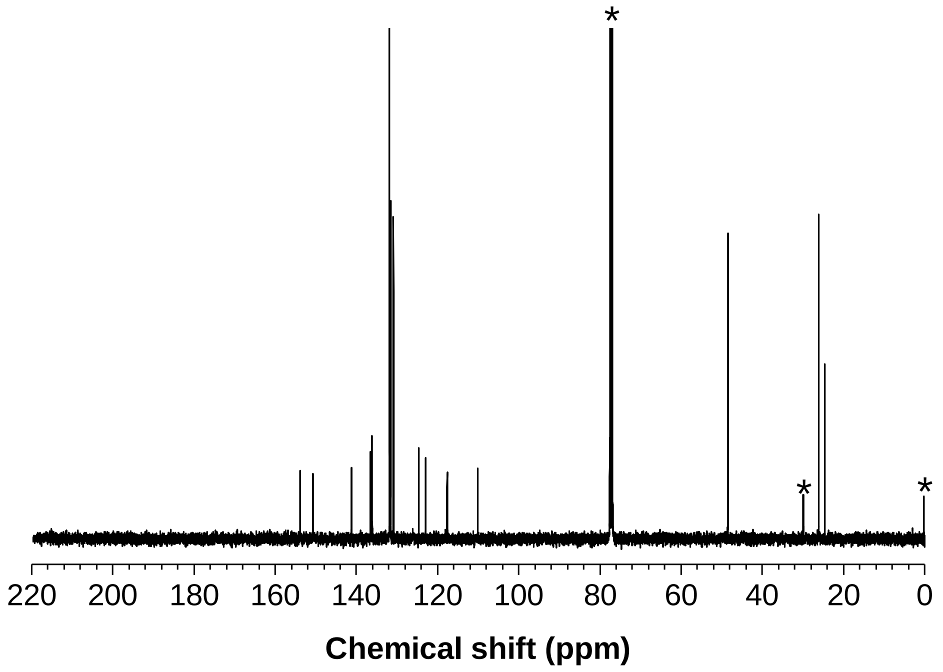

Figure S12. ^{13}C NMR spectrum of **Py-2** in $CDCl_3$. The solvent peaks are marked with asterisks.

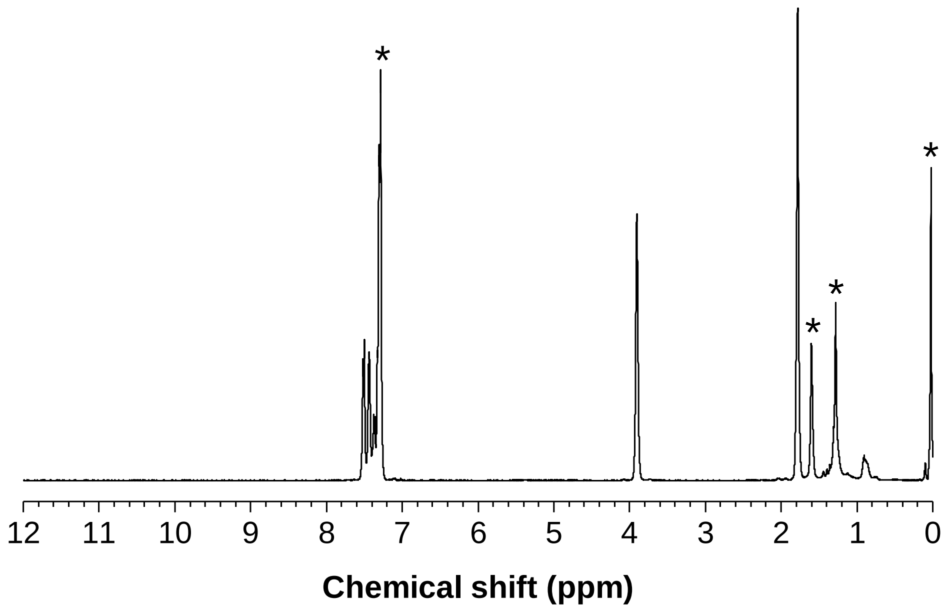

Figure S13. ^{1}H NMR spectrum of **Py-3** in $CDCl_3$. The solvent peaks are marked with asterisks.

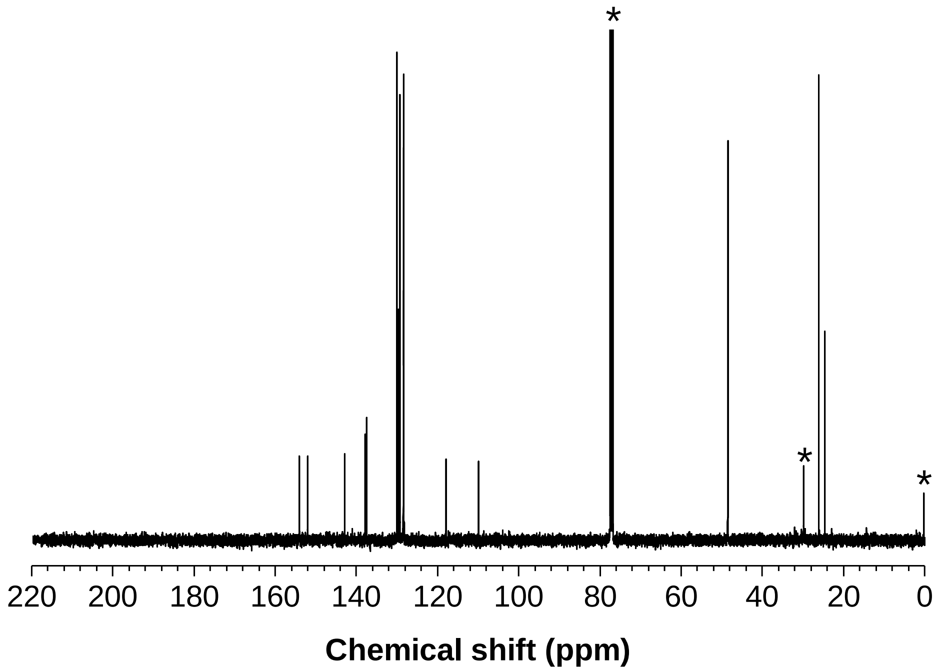

Figure S14. ^{13}C NMR spectrum of **Py-3** in $CDCl_3$. The solvent peaks are marked with asterisks.

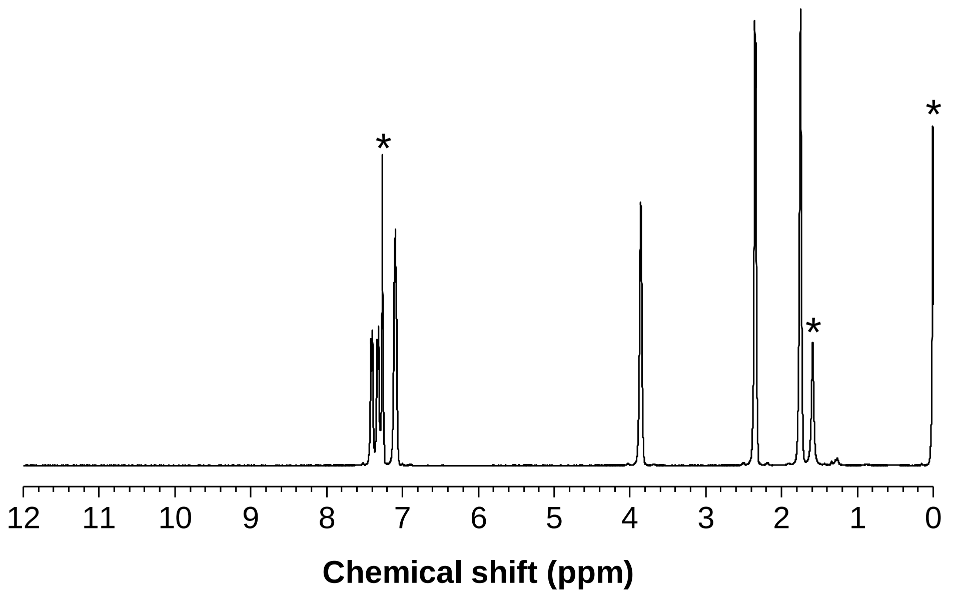

Figure S15. ^{1}H NMR spectrum of **Py-4** in $CDCl_3$. The solvent peaks are marked with asterisks.

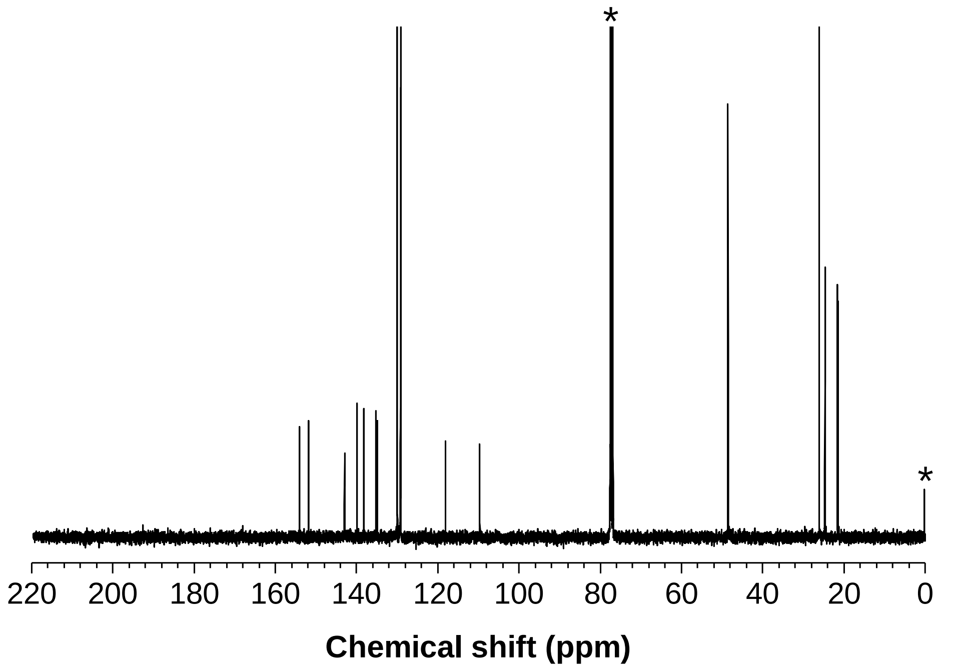

Figure S16. ^{13}C NMR spectrum of **Py-4** in $CDCl_3$. The solvent peaks are marked with asterisks.

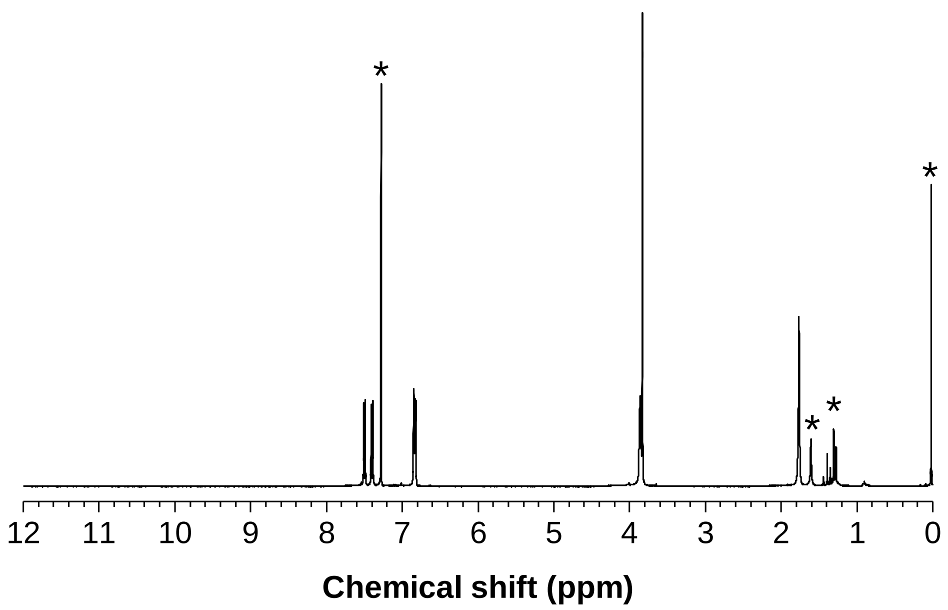

Figure S17. ^{1}H NMR spectrum of **Py-5** in $CDCl_3$. The solvent peaks are marked with asterisks.

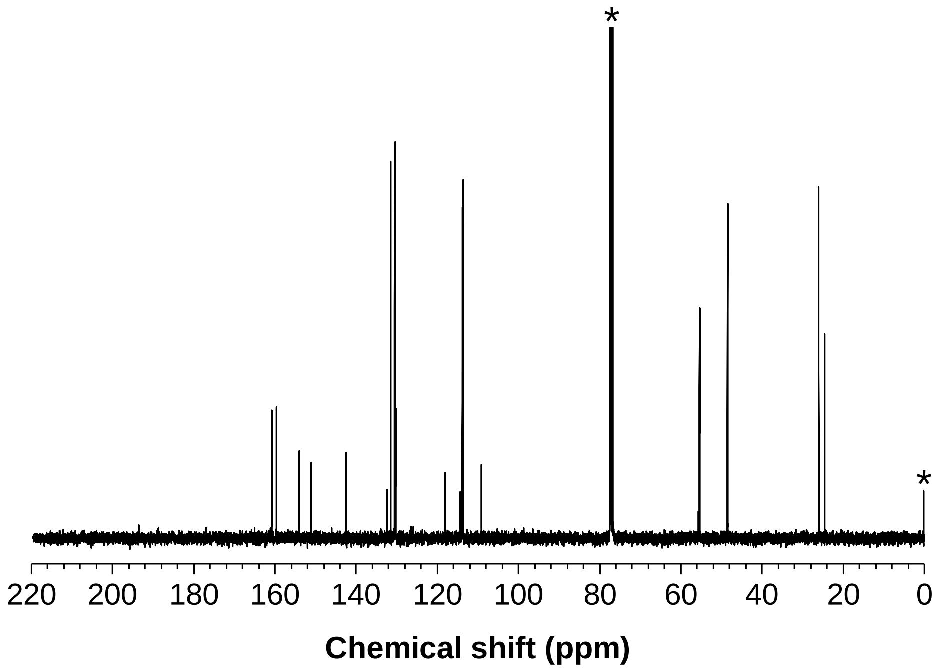

Figure S18. ^{13}C NMR spectrum of **Py-5** in $CDCl_3$. The solvent peaks are marked with asterisks.

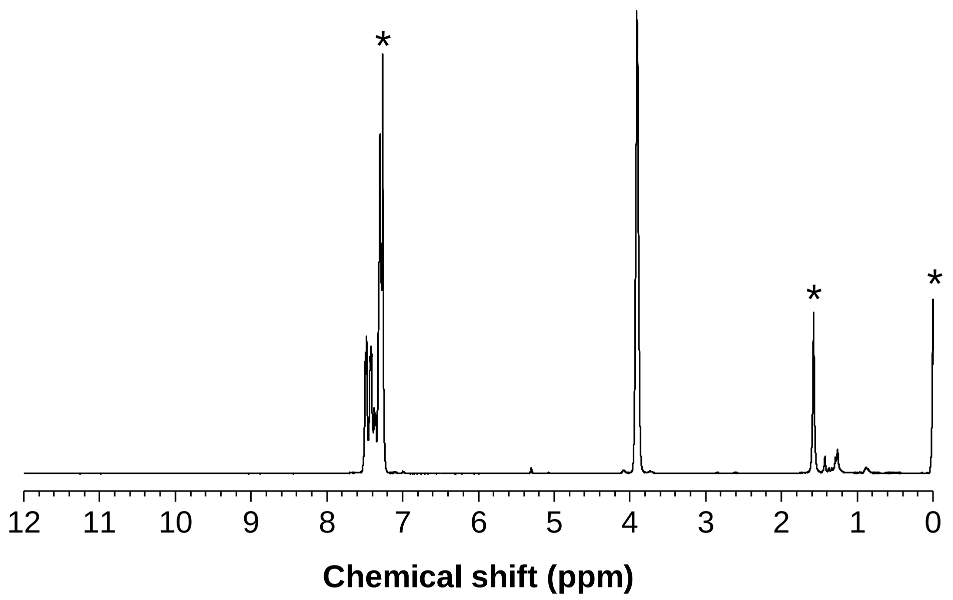

Figure S19. ^{1}H NMR spectrum of **Py-6** in $CDCl_3$. The solvent peaks are marked with asterisks.

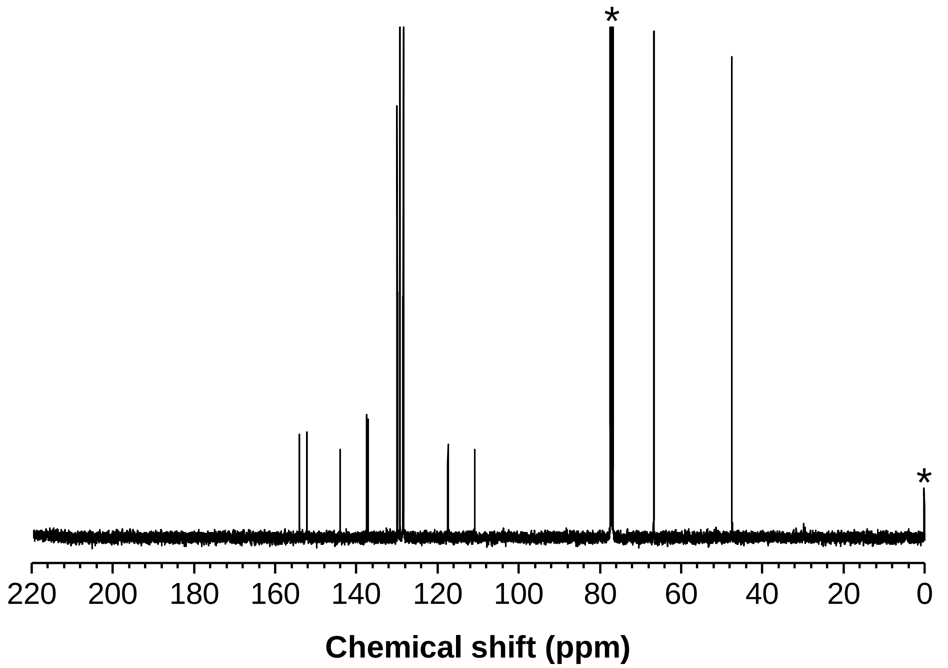

Figure S20. ^{13}C NMR spectrum of **Py-6** in $CDCl_3$. The solvent peaks are marked with asterisks.

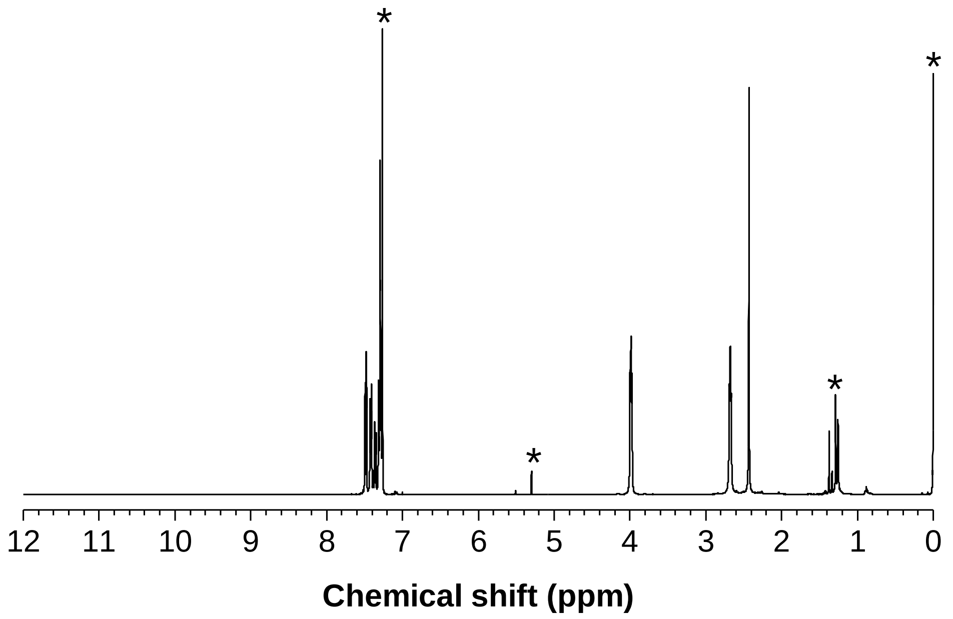

Figure S21. ^{1}H NMR spectrum of **Py-7** in $CDCl_3$. The solvent peaks are marked with asterisks.

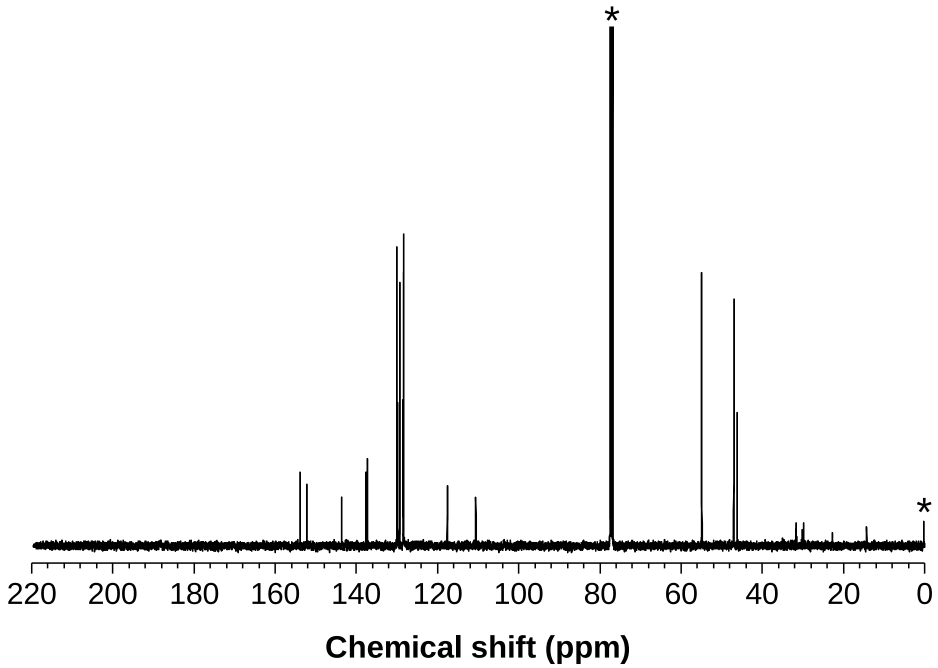

Figure S22. ^{13}C NMR spectrum of **Py-7** in $CDCl_3$. The solvent peaks are marked with asterisks.

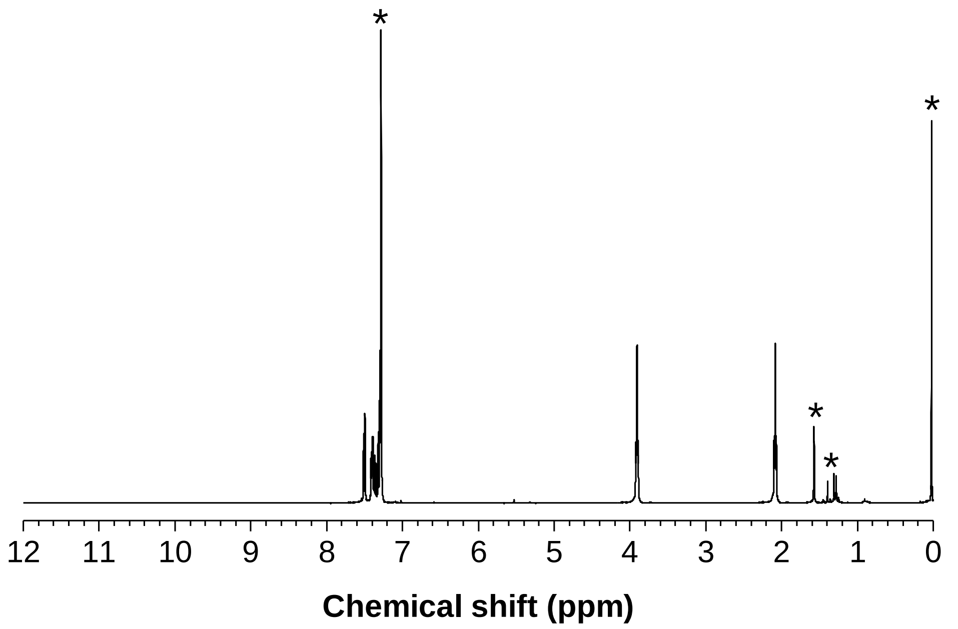

Figure S23. ^{1}H NMR spectrum of **Py-8** in $CDCl_3$. The solvent peaks are marked with asterisks.

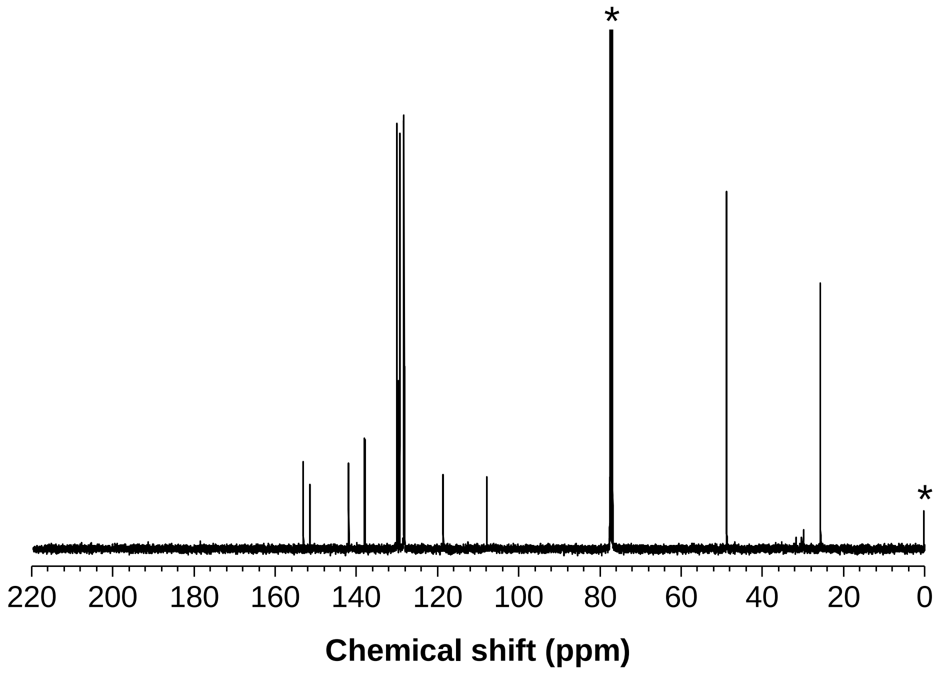

Figure S24. ^{13}C NMR spectrum of **Py-8** in $CDCl_3$. The solvent peaks are marked with asterisks.

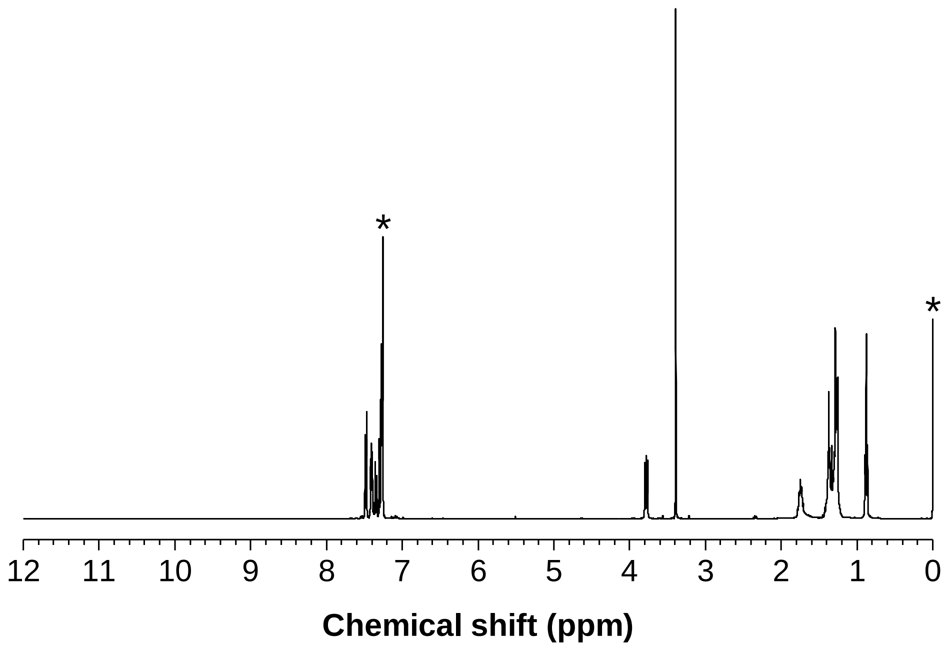

Figure S25. ^{1}H NMR spectrum of **Py-9** in $CDCl_3$. The solvent peaks are marked with asterisks.

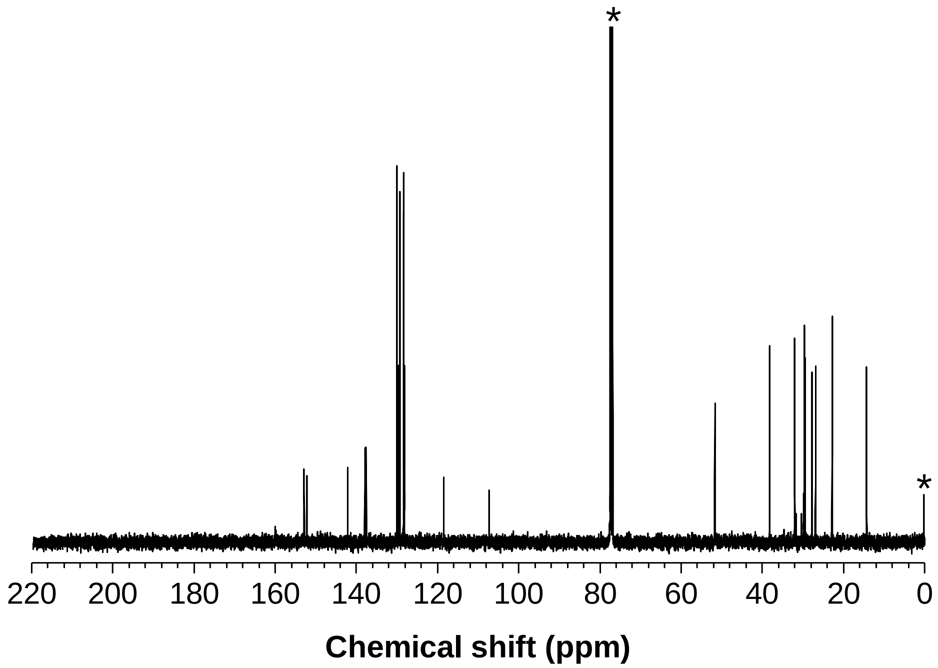

Figure S26. ^{13}C NMR spectrum of **Py-9** in $CDCl_3$. The solvent peaks are marked with asterisks.

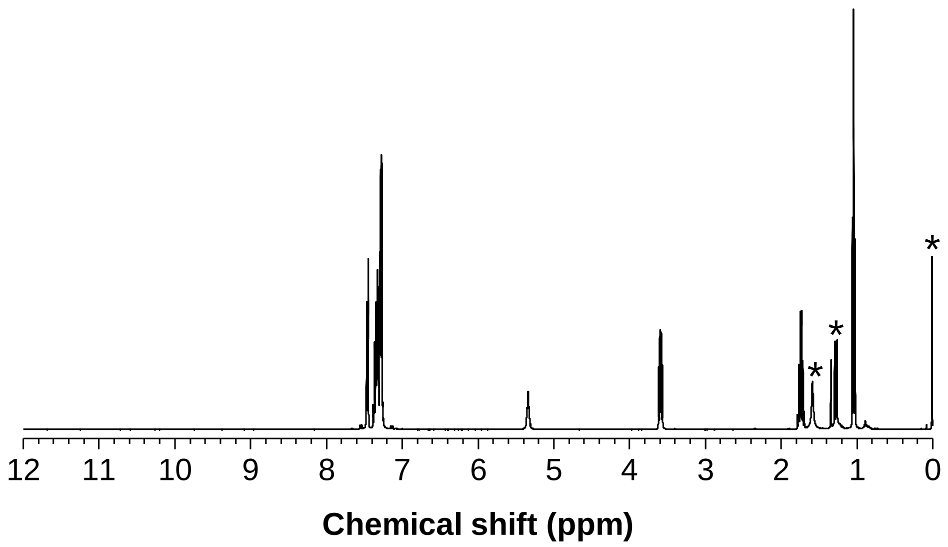

Figure S27. ^{1}H NMR spectrum of **Py-10** in $CDCl_3$. The solvent peaks are marked with asterisks.

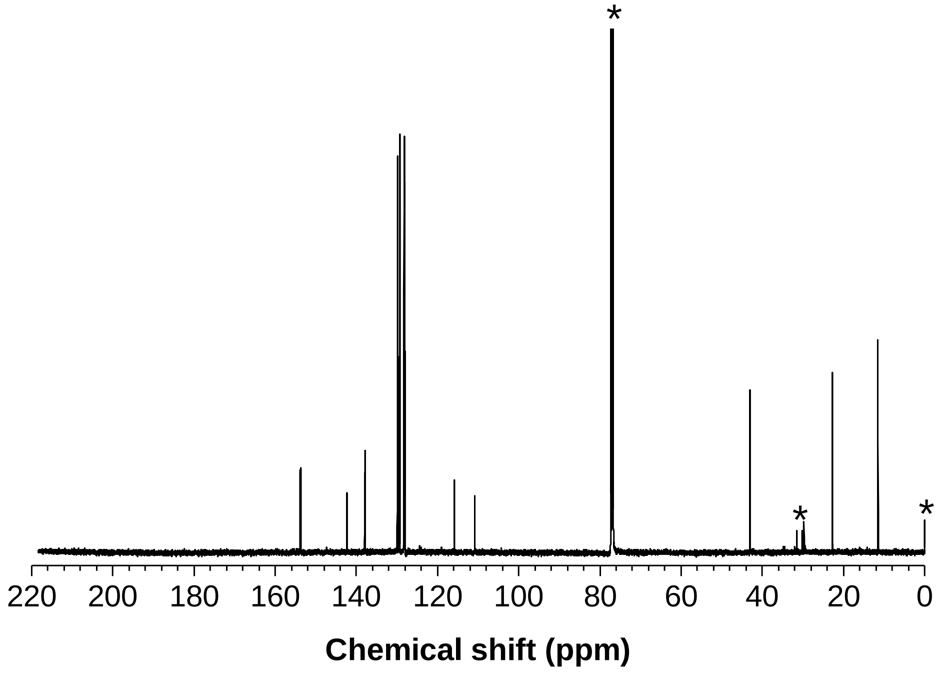

Figure S28. ^{13}C NMR spectrum of **Py-10** in $CDCl_3$. The solvent peaks are marked with asterisks.

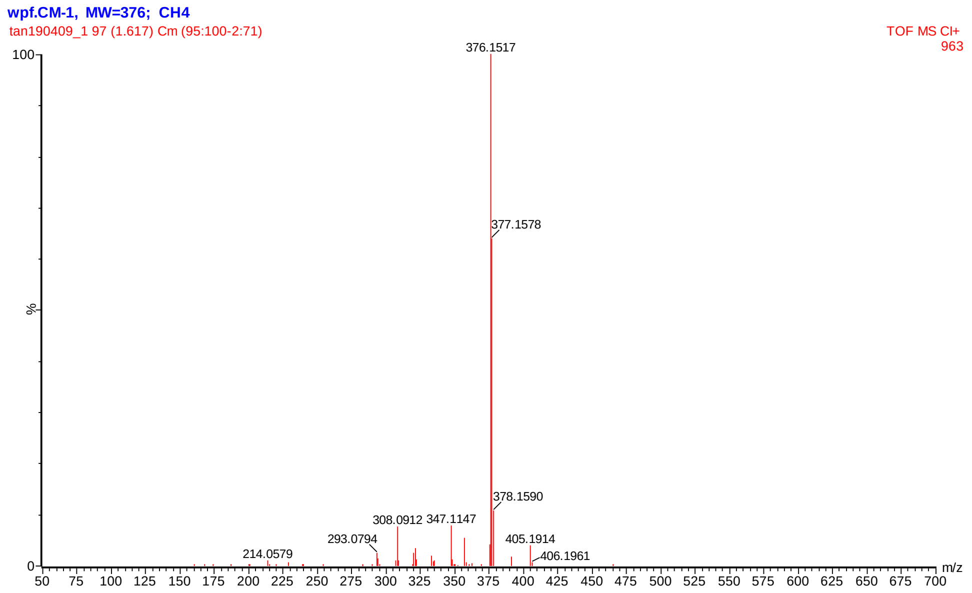

Figure S29. HRMS of **Py-1**.

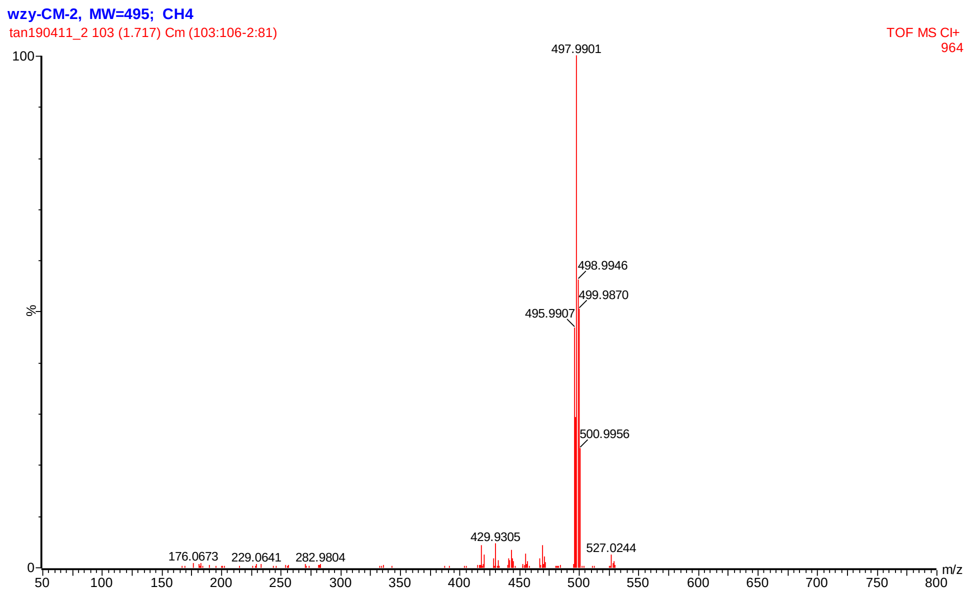

Figure S30. HRMS of **Py-2**.

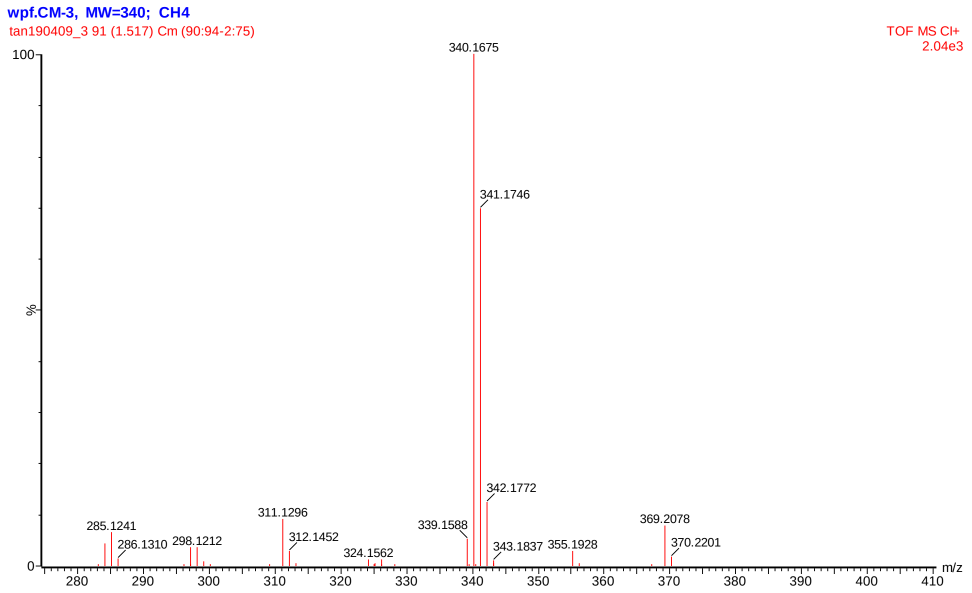

Figure S31. HRMS of **Py-3**.

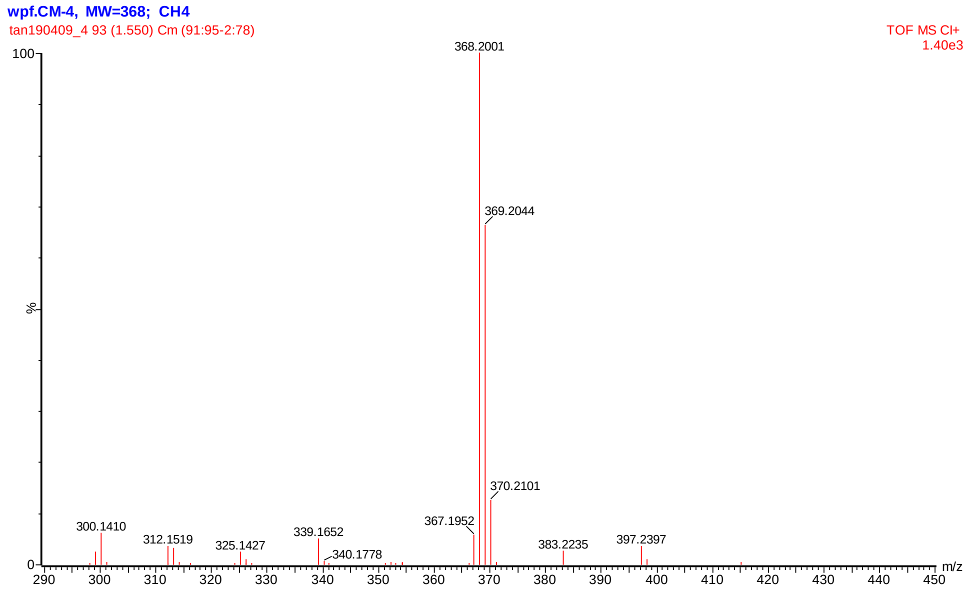

Figure S32. HRMS of **Py-4**.

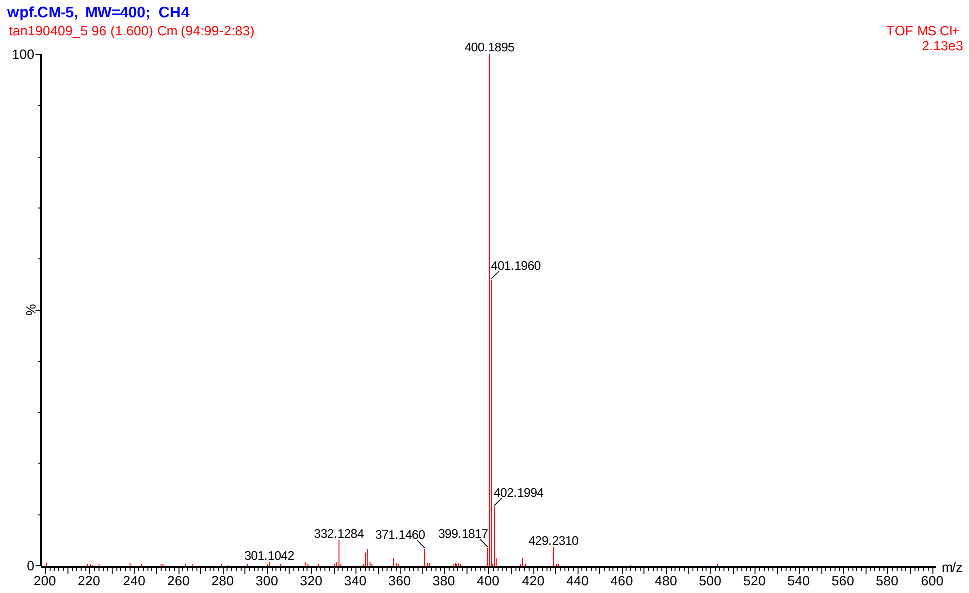

Figure S33. HRMS of **Py-5**.

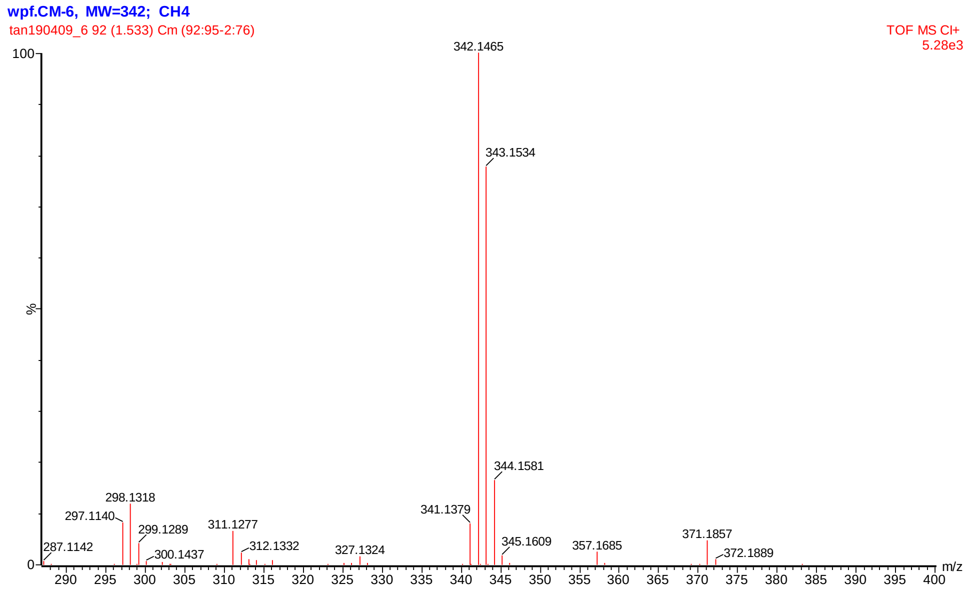

Figure S34. HRMS of **Py-6**.

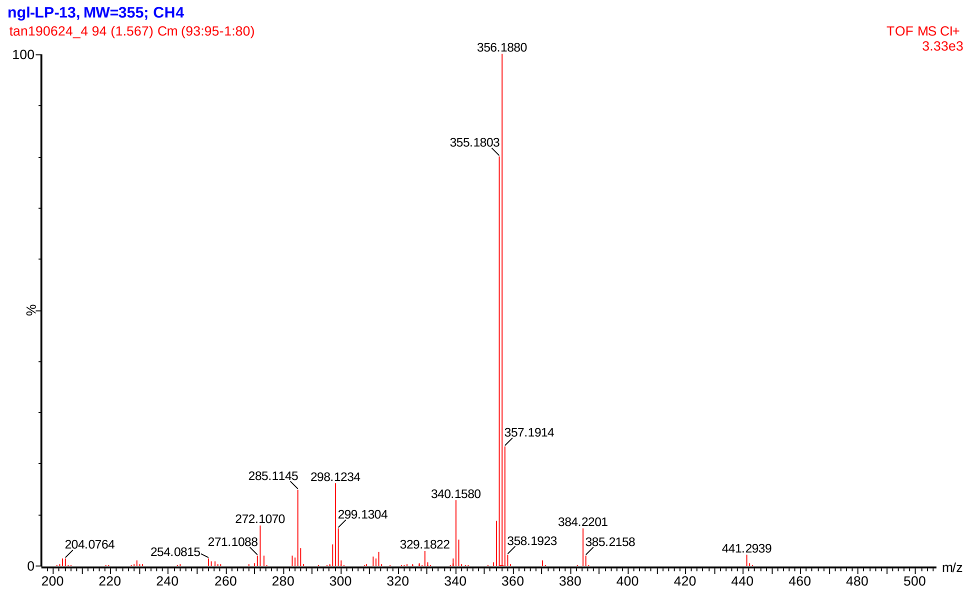

Figure S35. HRMS of **Py-7**.

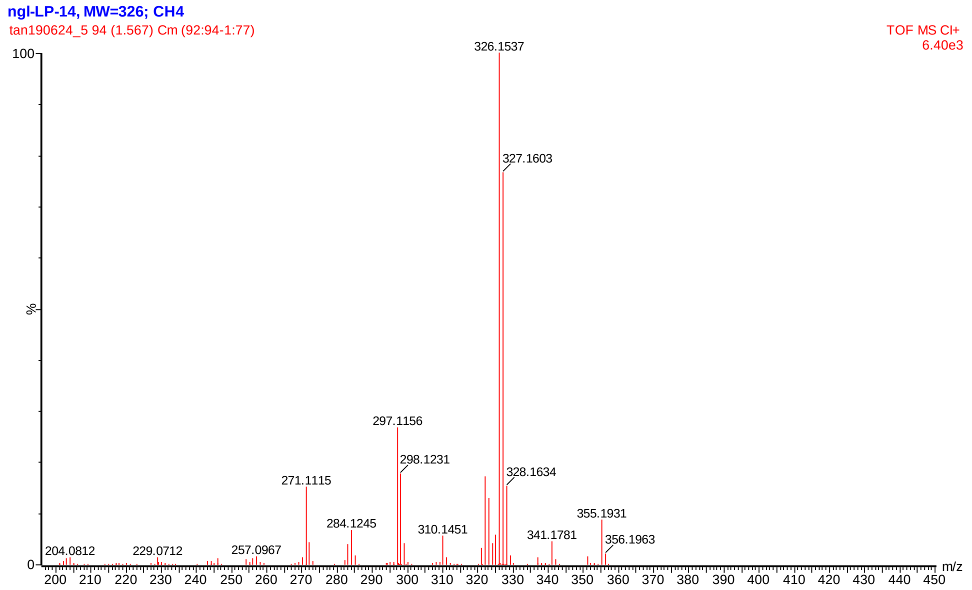

Figure S36. HRMS of **Py-8**.

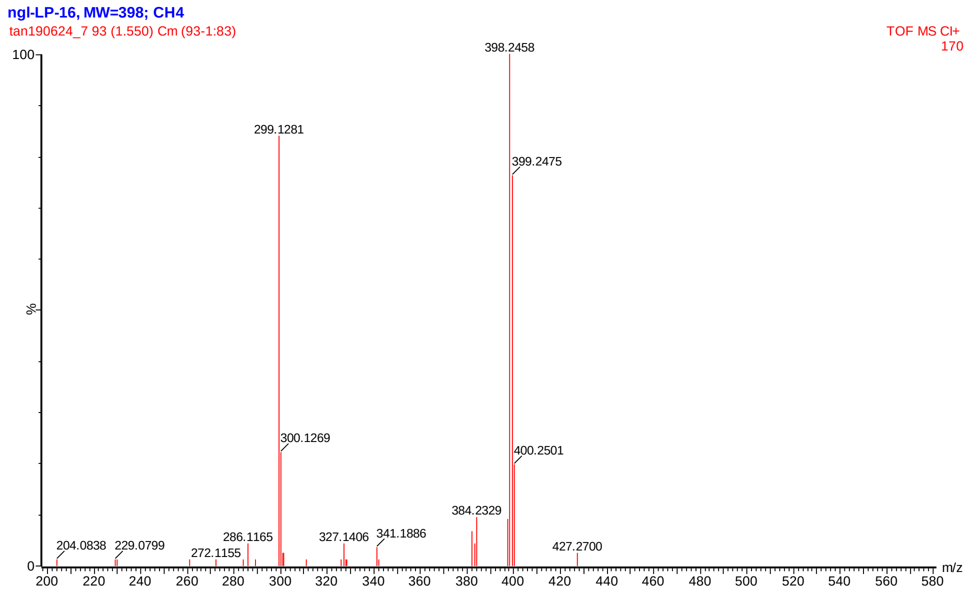

Figure S37. HRMS of **Py-9**.

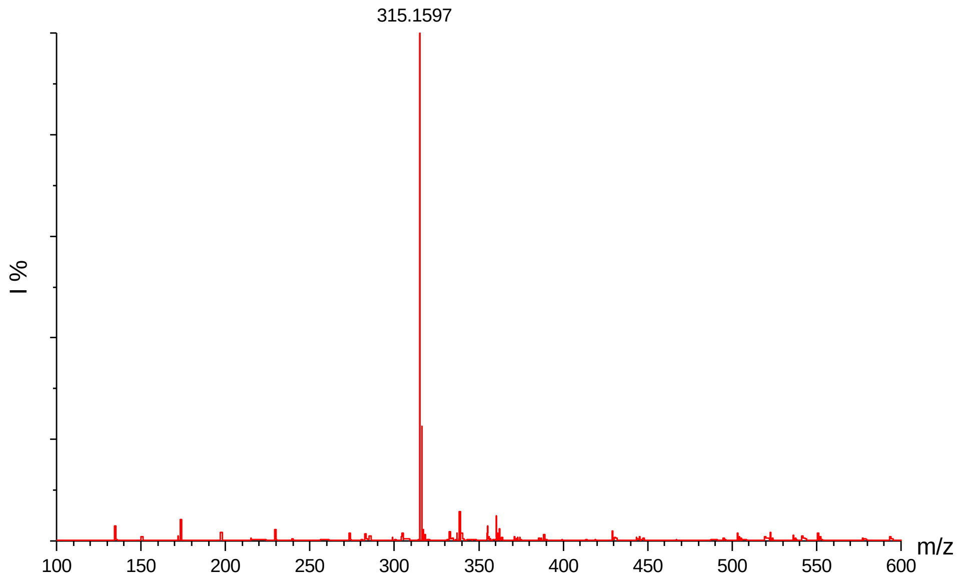

Figure S38. HRMS of **Py-10**.

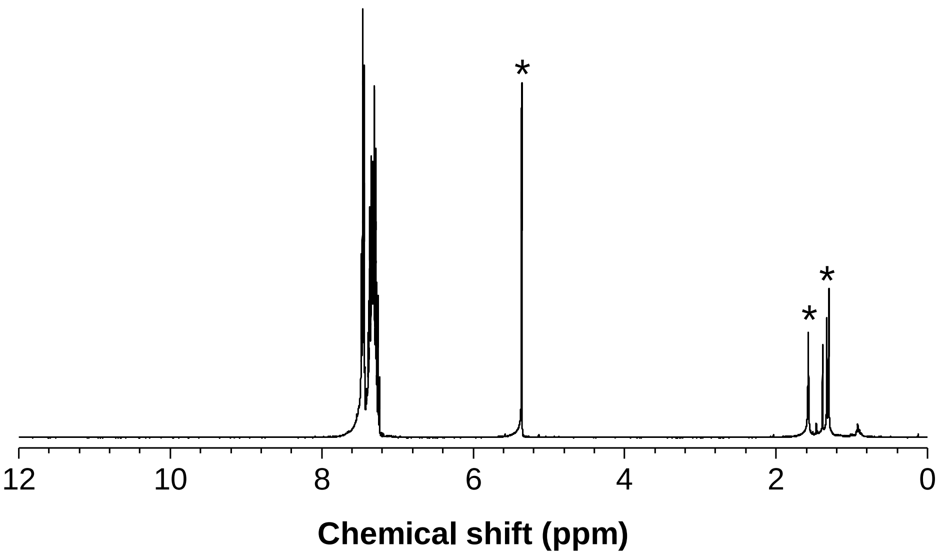

Figure S39. ^{1}H NMR spectrum of **Py-DPA** in CD_2Cl_2. The solvent peaks are marked with asterisks.

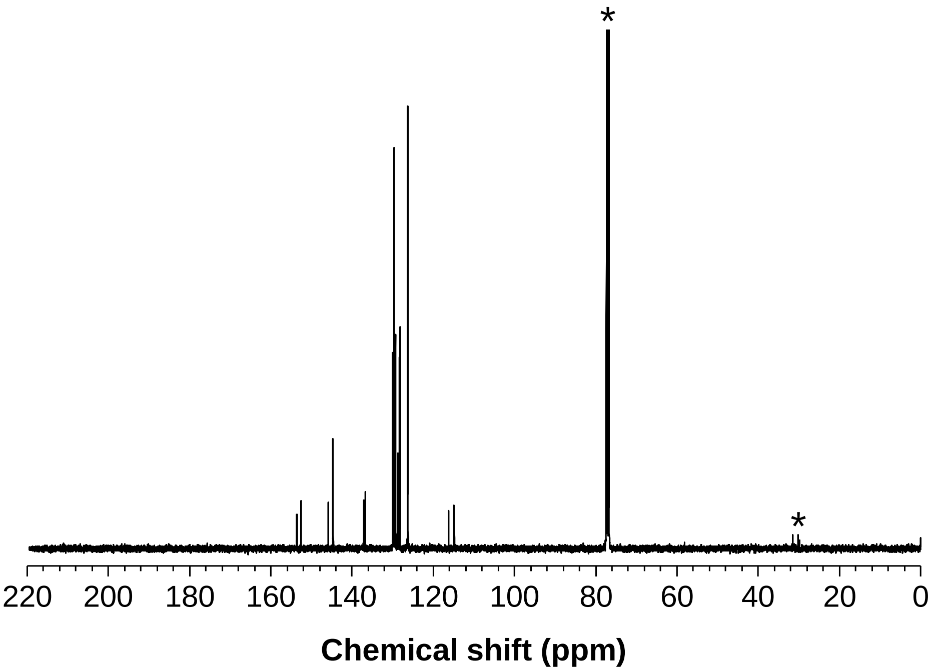

Figure S40. ^{13}C NMR spectrum of **Py-DPA** in $CDCl_3$. The solvent peaks are marked with asterisks.

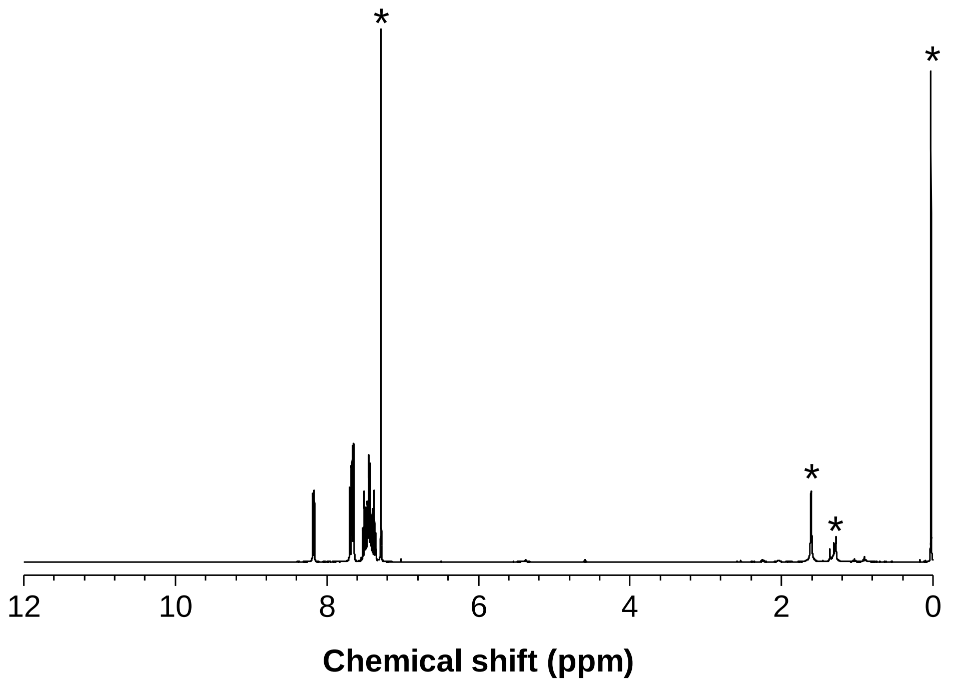

Figure S41. ^{1}H NMR spectrum of **Py-Cz** in $CDCl_3$. The solvent peaks are marked with asterisks.

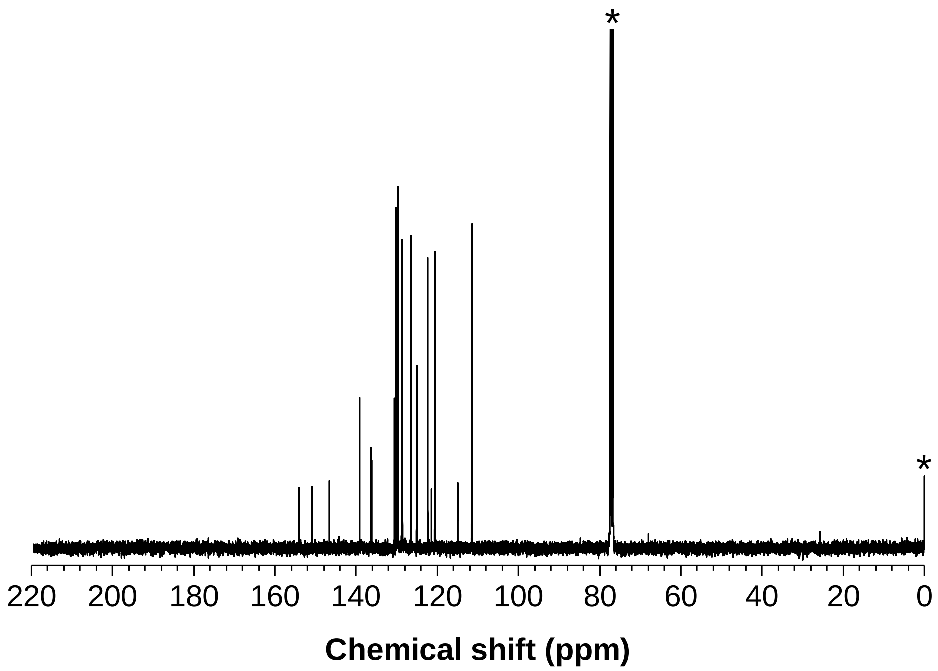

Figure S42. ^{13}C NMR spectrum of **Py-Cz** in $CDCl_3$. The solvent peaks are marked with asterisks.

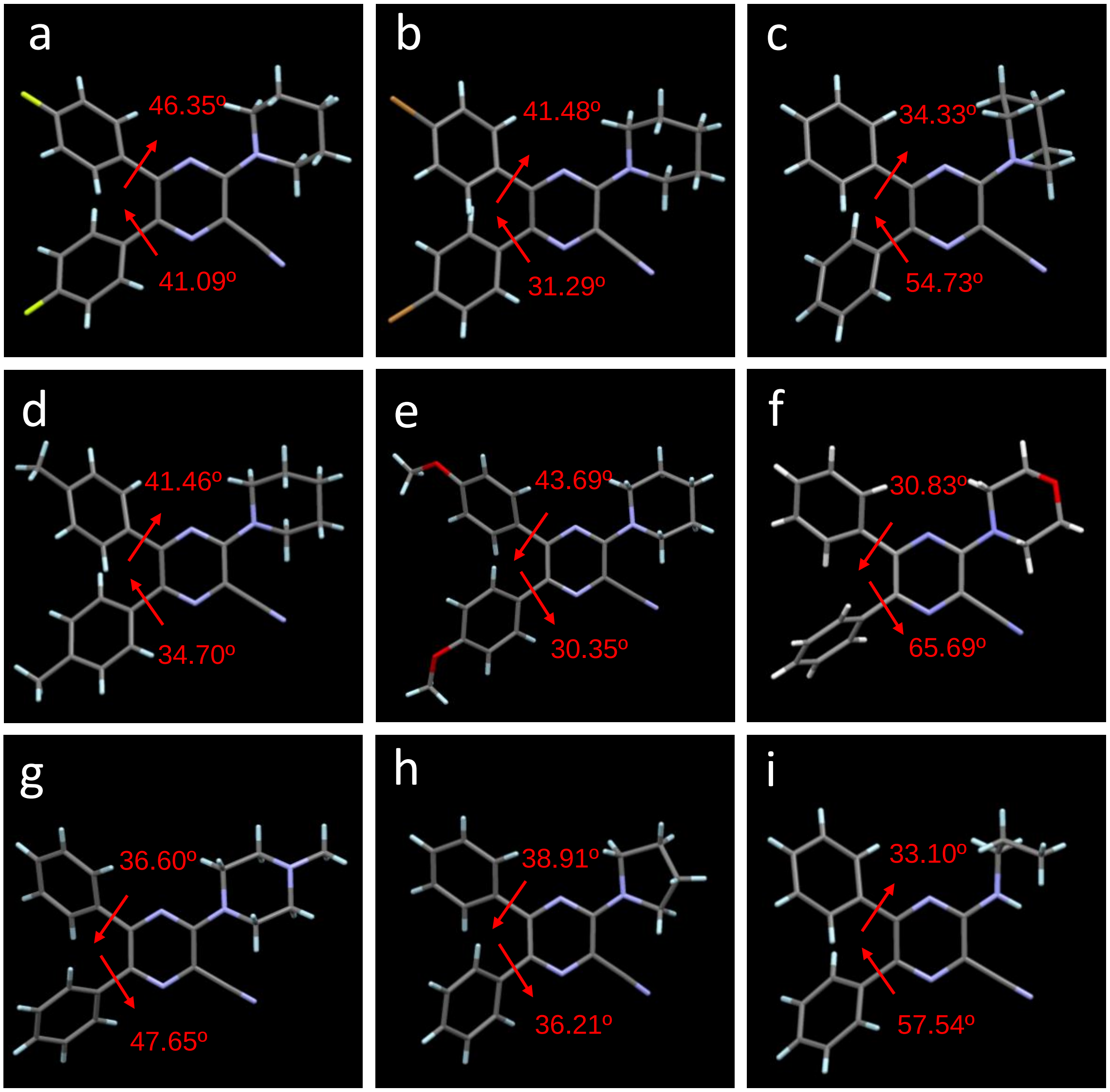

Figure S43. Crystal structures of a) **Py-1**, b) **Py-2**, c) **Py-3**, d) **Py-4**, e) **Py-5**, f) **Py-6**, g) **Py-7**, h) **Py-8** and i) **Py-10**, and the dihedral angles between the surrounding phenyl rings and central pyrazine ring. The rotation direction of phenyl rings is marked with arrow.

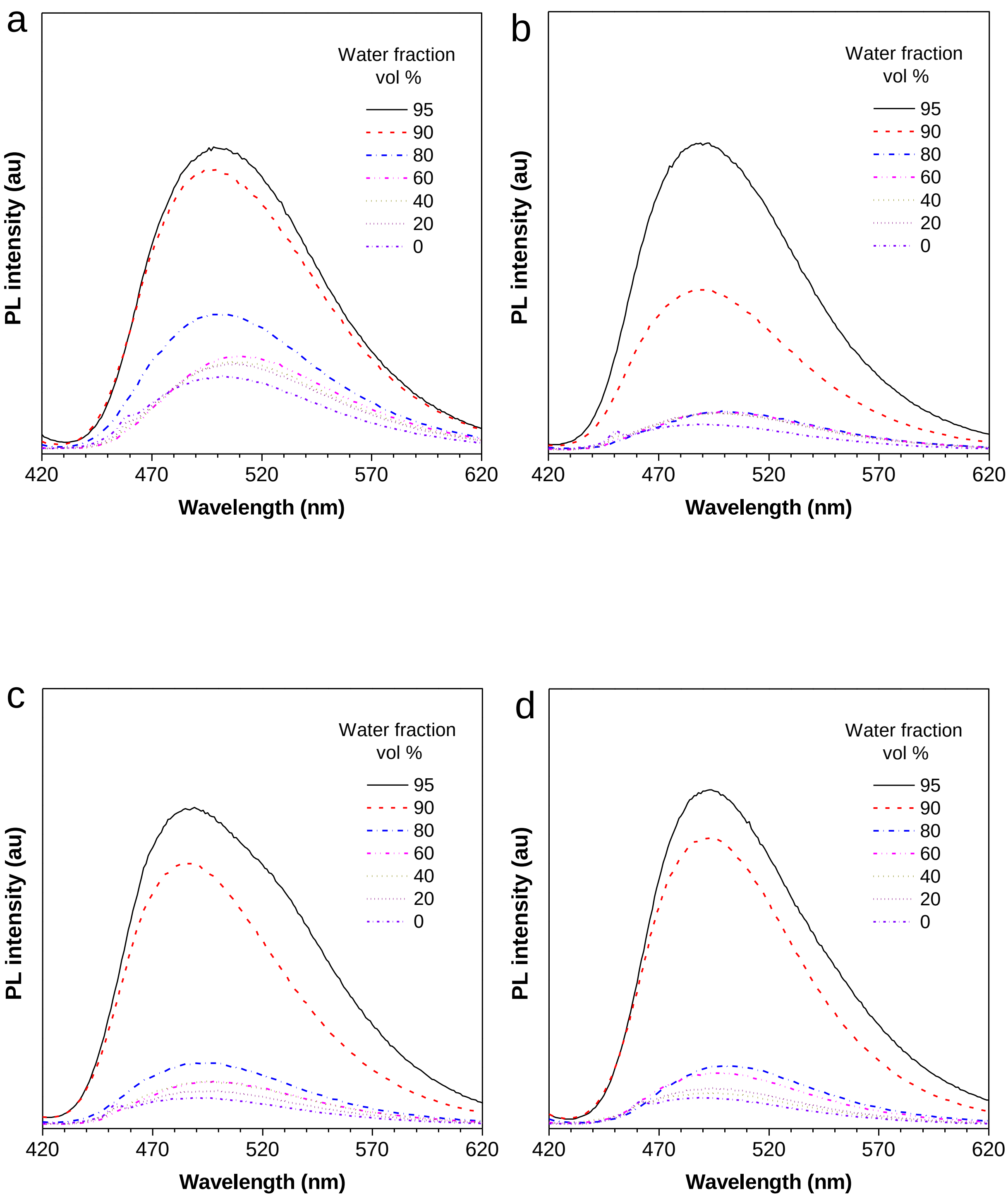
a
b
c
d
Water fraction
vol %
95
90
80
60
40
20
0
PL intensity (au)
Wavelength (nm)
420
470
520
570
620

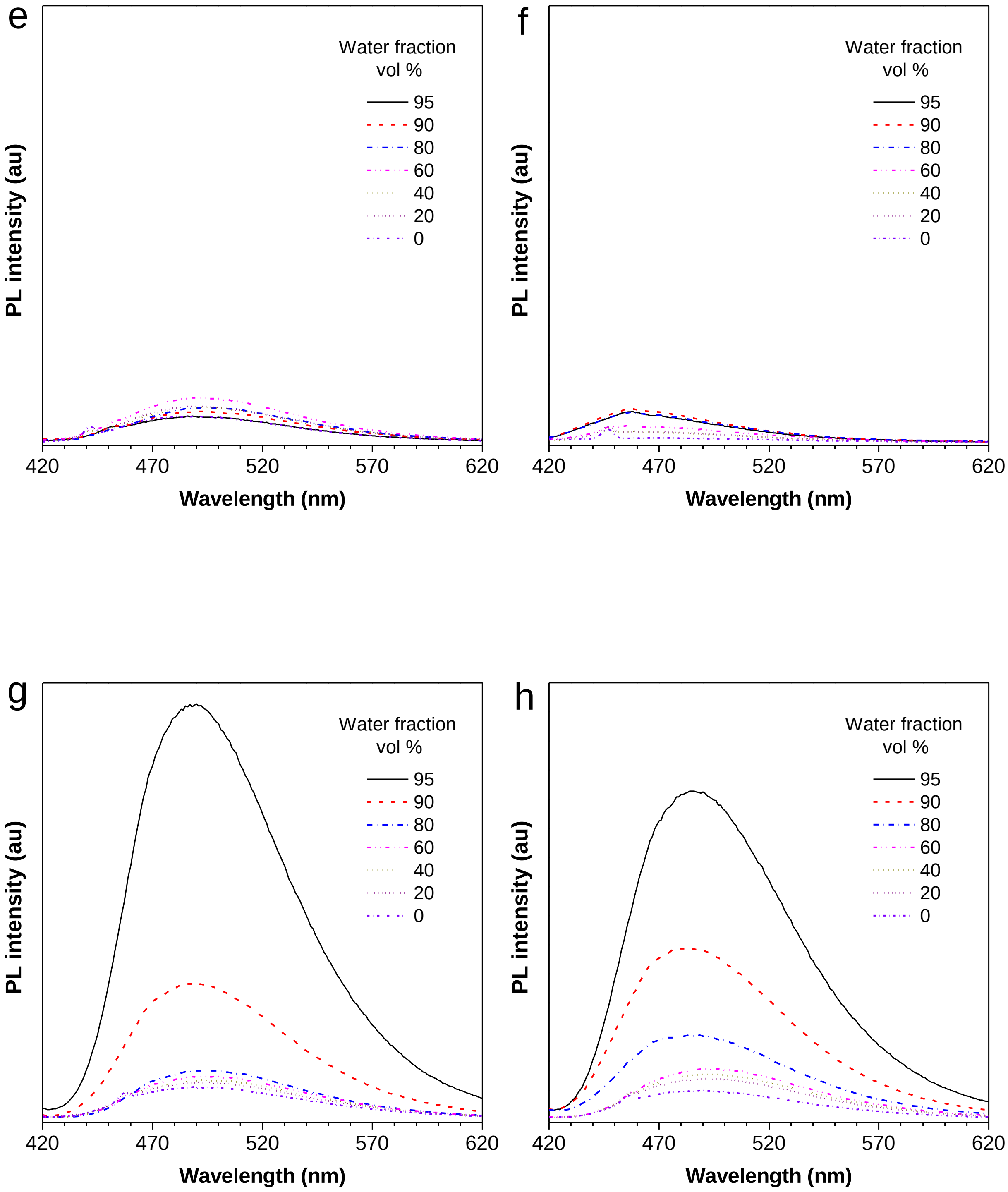

e
f
g
h
Water fraction vol %
95
90
80
60
40
20
0
PL intensity (au)
Wavelength (nm)
420
470
520
570
620

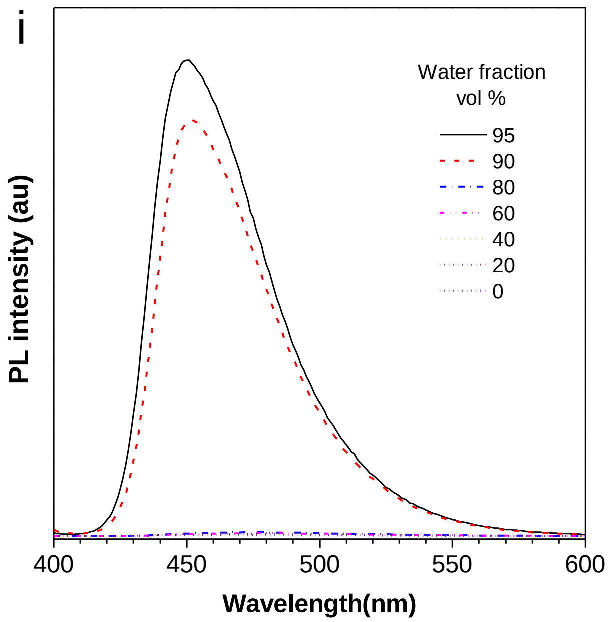

Figure S44. PL spectra of a) **Py-2**, b) **Py-3**, c) **Py-4**, d) **Py-5**, e) **Py-6**, f) **Py-7**, g) **Py-8**, h) **Py-9** and i) **Py-10** in THF/water mixtures with different f_w, [AIEgen] = 10 μM.

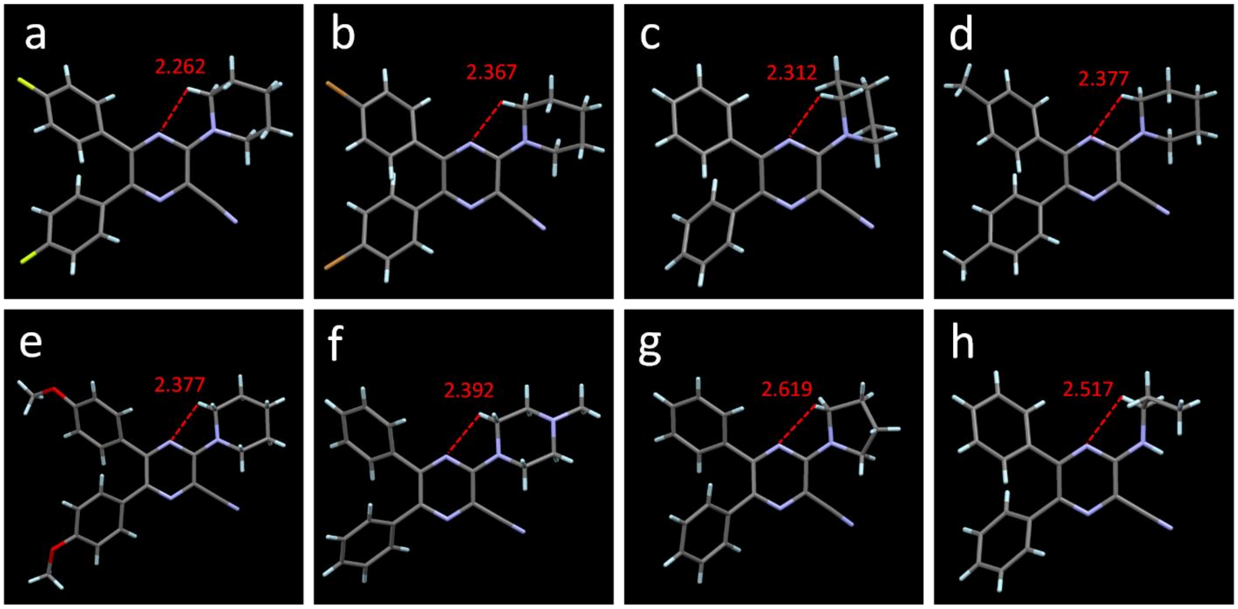

Figure S45. The intramolecular C-H⋯N interaction in a) **Py-1**, b) **Py-2**, c) **Py-3**, d) **Py-4**, e) **Py-5**, f) **Py-7**, g) **Py-8** and h) **Py-10** with indicated distances (Å).

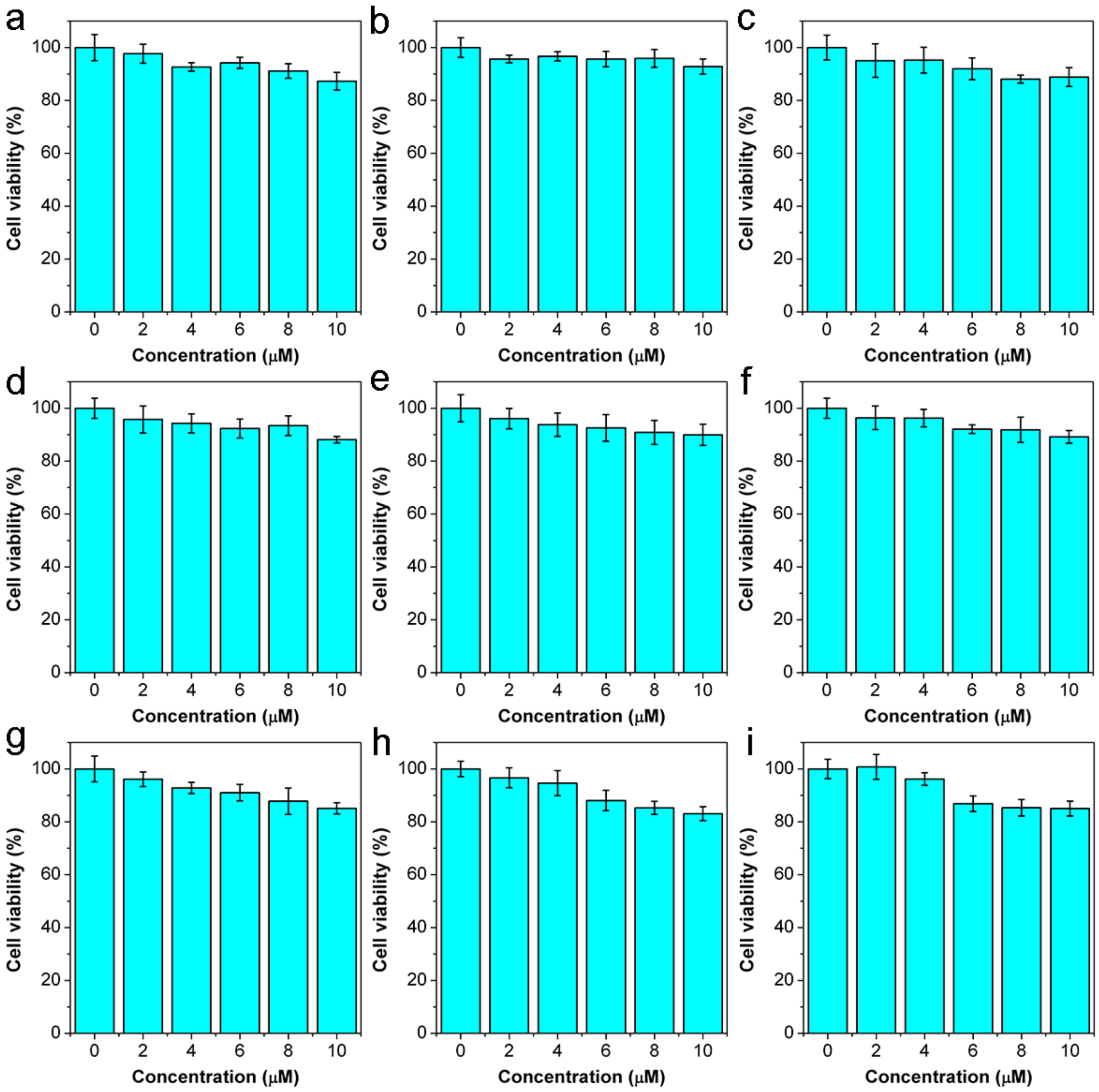

Figure S46. Cytotoxicity of a) **Py-1**, b) **Py-2**, c) **Py-3**, d) **Py-4**, e) **Py-5**, f) **Py-6**, g) **Py-7**, h) **Py-8**, and i) **Py-9** in HeLa cells.

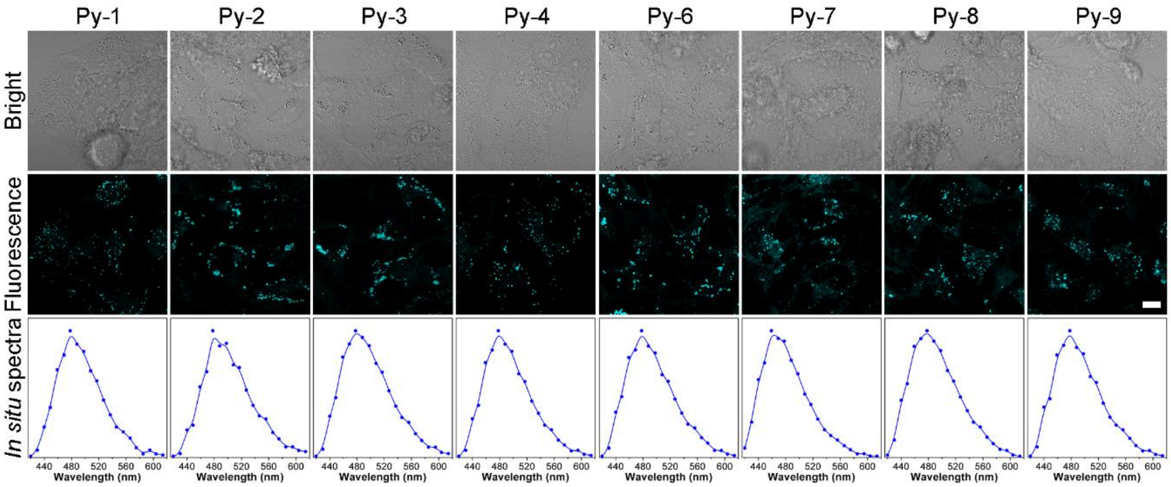

Figure S47. Confocal laser scanning microscopy images of HeLa cells stained with **Py-1**, **Py-2**, **Py-3**, **Py-4**, **Py-6**, **Py-7**, **Py-8**, and **Py-9** (1 μM) and their *in situ* fluorescence spectra. Scale bar: 10 μm.

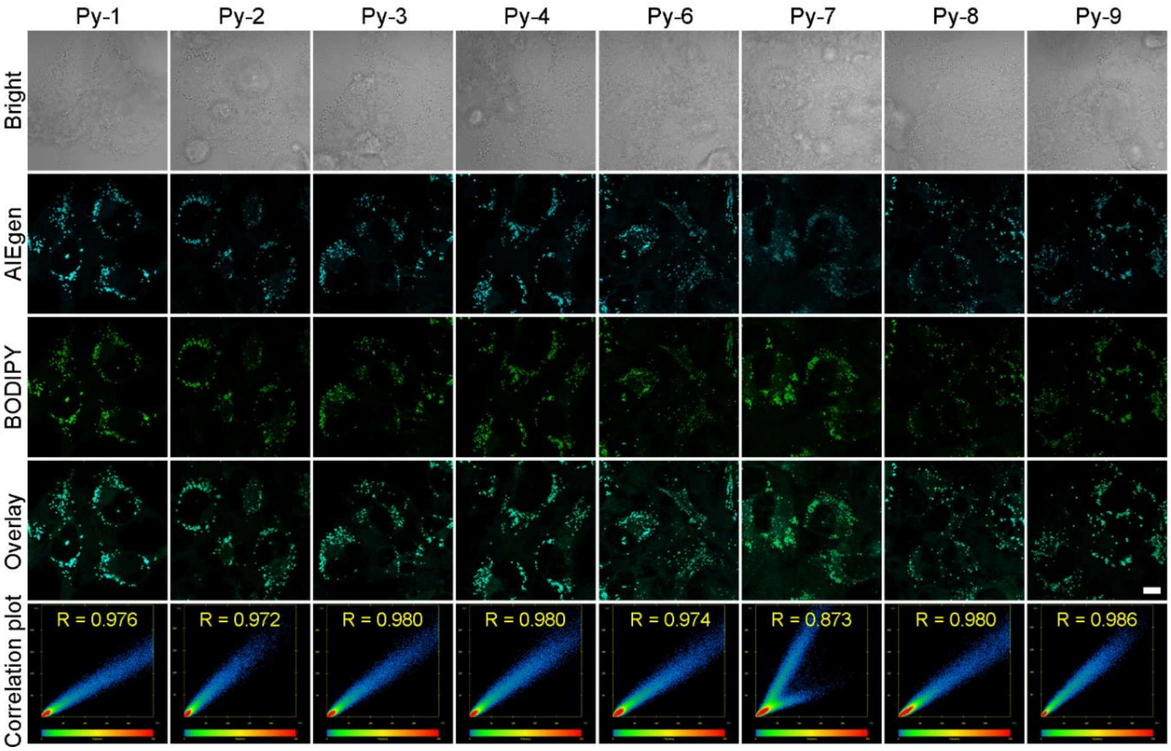

Figure S48. Confocal laser scanning microscopy images of HeLa cells stained with AIEgens (**Py-1**, **Py-2**, **Py-3**, **Py-4**, **Py-6**, **Py-7**, **Py-8**, and **Py-9**, 1 μM) and BODIPY 493/503 (0.2 μM), and their fluorescence intensity correlation plots of AIEgens and BODIPY 493/503. R is the Pearson's coefficient. Scale bar: 10 μm.

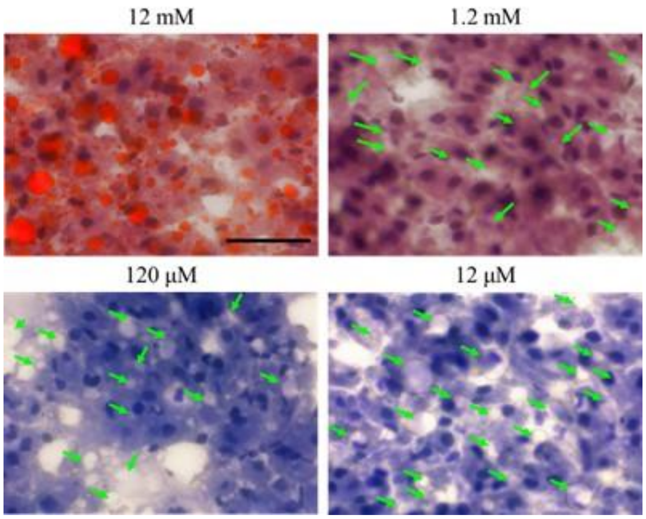

Figure S49. Bright-field images of the high-fat feeding guinea pig liver tissue stained with Oil Red O at different concentrations. The nuclei were stained hematoxylin. Green arrows indicate lipid droplets. Scale bar: 40 μm.

Table S1. Amine scope for the reaction and preparation of AIEgens.[a)]

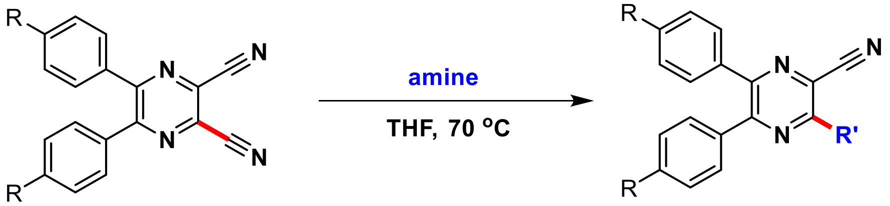

entry	amine	R	R’	time [h]	yield [%]
1 (**Py-1**)	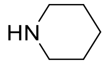	F	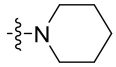	17	93
2 (**Py-2**)	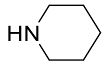	Br	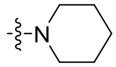	22	86.6
3 (**Py-3**)	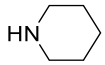	H	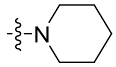	18	91.2
4 (**Py-4**)	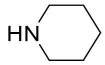	Me	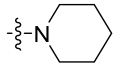	24	75.8
5 (**Py-5**)	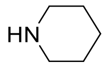	OMe	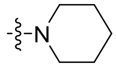	14	84.2
6 (**Py-6**)	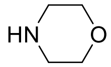	H	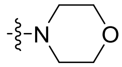	35	99.8
7 (**Py-7**)	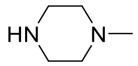	H	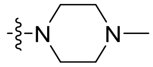	14	83.5
8 (**Py-8**)	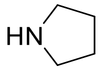	H	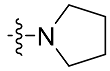	18	93.1
9 (**Py-9**)	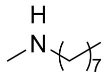	H	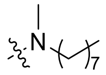	8	18.4
10 (**Py-10**)	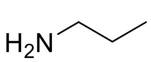	H	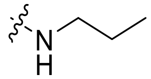	10	40.4
11 (**Py-DPA**)	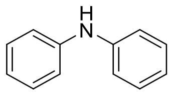	H	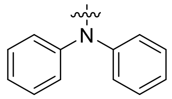	24	87.8
12 (**Py-Cz**)	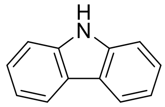	H	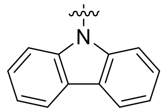	24	36.9

[a)] The reactions are carried out at 70 ℃ with nitrile-substituted pyrazines and amines (mol ratio = 1: 1.2 for entries 1-5 and 8, and 1: 10 for entries 6, 7 and 9-11) in THF in the air. For entries 12 and 13, the reactions are carried out with aryl amines (1.2 equiv.) deprotonated by the n-BuLi.

Table S2. Photo-physical properties of AIEgens.

Compound	$\lambda_{abs,soln}$ [a] (nm)	λ_{em} (nm)		Φ_F [c] (%)		α_{AIE} [d]	τ [e] (ns)		k_r [f] (10^8 S^{-1})		k_{nr} [g] (10^8 S^{-1})	
		Soln[a]	Aggr[b]	Soln[a]	Aggr[b]		Soln[a]	Aggr[b]	Soln[a]	Aggr[b]	Soln[a]	Aggr[b]
**Py-1**	399	488	486	1.43	23.8	17	0.46	8.7	0.31	0.27	21.4	0.88
**Py-2**	403	502	508	2.43	8.17	3	0.56	1.82	0.43	0.45	17.4	5.05
**Py-3**	397	490	479	1.60	30.7	19	0.41	6.47	0.39	0.48	24	1.07
**Py-4**	399	492	504	2.03	24.6	12	0.41	5.19	0.5	0.47	23.9	1.45
**Py-5**	405	488	496	2.60	8.53	3	0.45	1.62	0.58	0.53	21.6	5.65
**Py-6**	390	488	469	1.33	70.5	53	0.43	12.4	0.31	0.57	23	0.24
**Py-7**	394	468	481	0.5	3.97	8	0.51	1.51	0.1	0.26	19.5	6.36
**Py-8**	402	492	493	1.67	15.4	9	0.4	3.17	0.42	0.49	24.6	2.67
**Py-9**	401	490	493	0.6	10.6	18	0.45	2.44	0.13	0.43	22.1	3.66
**Py-10**	393	472	461	0.4	43.0	108	0.48	6.7	0.08	0.64	20.8	0.85
**Py-DPA**	405	544	523	2.8	18.5	7	5.13	5.10	0.06	0.36	1.90	1.60
**Py-Cz**	330	510	476	12.6	35.1	3	8.87	6.01	0.14	0.58	0.99	1.08

[a] In THF solution (10^{-5} M); [b] In powder except for Py-9 in film; [c] Absolute quantum yield determined by an integrating sphere; [d] Ratio between the Φ_F of molecules in the aggregate and solution state; [e] Fluorescence lifetime measured at room temperature in air; [f] Rate of radiative decay determined by Φ_F/τ; [g] Rate of non-radiative decay determined by $(1- \Phi_F)/\tau$.

Reference

[1] A. Qin, J. W. Y. Lam, F. Mahtab, C. K. W. Jim, L. Tang, J. Sun, H. H. Y. Sung, I. D. Williams, B. Z. Tang, *Appl. Phy. Lett.*, 2009, **94**, 253308.

[2] F. Neese, *WIREs Comput. Mol. Sci.*, 2012, **2**, 73–78.

[3] F. Neese, *WIREs Comput. Mol. Sci.*, 2018, **8**, e1327.

[4] A. V. Marenich, C. J. Cramer, D. G. Truhlar, *J. Phys. Chem. B*, 2009, **113**, 6378–6396.

[5] S. Grimme, J. Antony, S. Ehrlich, H. Krieg, *J. Chem. Phys.*, 2010, **132**, 154104.

[6] M. Frisch, G. Trucks, H. Schlegel, G. Scuseria, M. Robb, J. Cheeseman, G. Scalmani, V. Barone, G. Petersson, H. Nakatsuji, et al. Gaussian 16, Revision A.03, Gaussian, Inc., Wallingford CT, 2016.

[7] Z. Shuai, *Chin. J. Chem.*, 2020, **38**, 1223–1232.

[8] Z. Shuai, Peng, Q. *Phys. Rep.*, 2014, **537**, 123–156.

[9] Z. Shuai, Peng, Q. *Nat. Sci. Rev.*, 2017, **4**, 224–239.

[10] H. Park, S. Li, G. Niu, H. Zhang, Z. Song, Q. Lu, J. Zhang, C. Ma, R. T. K. Kwok, J. W. Y. Lam, K. S. Wong, X. Yu, Q. Xiong, B. Z. Tang, *Mater. Chem. Front.*, 2021, **5**, 1853–1862.

[11] J. Liang, S. Chen, Y. Hu, Y. Yang, J. Yuan, Y. Wu, S. Li, J. Lin, L. He, S. Hou, *Int. J. Biol. Macromol.*, 2018, **107**, 2201–2210.
